# The Impact of COVID-19 and Associated Interventions on Mental Health: A Cross-Sectional Study in a Sample of University Students

**DOI:** 10.3389/fpsyt.2021.801859

**Published:** 2022-01-26

**Authors:** Christina Camilleri, Cole S. Fogle, Kathryn G. O'Brien, Stephen Sammut

**Affiliations:** Department of Psychology, Franciscan University of Steubenville, Steubenville, OH, United States

**Keywords:** COVID-19, mental health, depression, anxiety, stress, coping, impact of event, university students

## Abstract

**Background:**

Mental health issues have continued to rise globally, including among university students. The COVID-19 pandemic has exacerbated the previously existing and concerning problem. Given that coping mechanisms have been proposed to mediate the relationship between stressors and mental health, the aim of our cross-sectional study was to investigate the mediation of coping mechanisms on the relationship between the impact of the COVID-19 pandemic and mental health.

**Methods:**

University students (≥18 years old; *N* = 676; 31% male, 69% female) were administered an anonymous survey addressing current demographics, COVID-19 pandemic-related demographics, personal experiences, sources of stress and perceived effect on mental health, politics, sources of news/information, and various pre-validated scales addressing mental health (DASS-21), the impact of the COVID-19 pandemic (IES-R) and coping strategies utilized (Brief COPE).

**Results:**

Our results indicate a substantial proportion of our sample reporting scores in the severe and extremely severe DASS-21 categories, in addition to ~50% reporting a perceived deterioration in mental health relative to pre-COVID-19 pandemic. Moreover, a substantial proportion of students reported IES-R scores at levels where PTSD is of clinical concern. Alarmingly, a significant proportion of females (~15%) reported scores reflecting potential long-term PTSD-related implications. Females tended to be more severely impacted in all mental health measures. Mediation analysis indicated that while dysfunctional coping mediated the relationship between the impact of the event (COVID-19 pandemic) and all three mental health outcomes, overall, this was not the case with the positive coping strategies.

**Conclusion:**

Our study appears to indicate a reduced buffering influence on negative mental health outcomes by the positive coping mechanisms investigated in relation to the COVID-19 pandemic and secondary interventions implemented. While the findings of this study pertain specifically to university students, they corroborate the existing extensive body of research (from physiological to behavioral, preclinical to clinical) pertaining to the response associated with major stressful events at every level of society. In this regard, the findings imply the necessity for health and other authorities, tasked with safeguarding public well-being, to avoid reactive interventions that do not appropriately balance the risks and benefits, potentially exacerbating pre-existing psychopathologies and compromising social order.

## Introduction

Well-being is a concept that incorporates the physical, mental, and social aspects of health and does not simply indicate an absence of disease (1, 2). Mental health, also referred to as emotional well-being (3), “includes the presence of positive emotions and moods (e.g., contentment, happiness), the absence of negative emotions (e.g., depression, anxiety), satisfaction with life, fulfillment and positive functioning,” as well as the ability to maintain autonomy (2, 4). Globally, the past several decades have seen a significant decline in mental health in the general population (5, 6).

One group, which appears to be rather vulnerable to negative mental health and has been of special interest, most especially in the past 10 years, is the university student population (7–9). This is, in part, due to the significant and formative period of development (neurodevelopmental, social, etc.) experienced by this age group, with potential long-term implications into adulthood (10–13). This dynamic neurological, and thus behavioral, period of development is also characterized by an increased predisposition to the onset of various psychiatric disorders (14–16).

Psychological stress is defined as “a particular relationship between the person and the environment that is appraised by the person as taxing or exceeding his or her resources and endangering his or her well-being” (17). Psychological stress can have a significant impact on the predisposition, development and expression of mental disorders (18) and, as such, it is necessary to have an understanding of the potential sources of stress in order to aid in finding potential remedies to alleviate the resulting negative consequences. Various stressors have been reported to relate to the mental health problems observed within the university student population, including academic performance, transition from high school, leaving home, changes in social relationships (e.g., new friendships), financial difficulties, post-graduation plans, problematic internet use, sleep, diet, and exercise (19–28). However, one unifying characteristic that identifies most of these stressors and their impact is that, in general, they reflect, with some variation, experiences faced, at varying periods of life, by a broad and significant proportion of the global population.

These challenges, however, bear a definite distinction from significant major external events over which the general population has little control, or those that have an impact not only at the personal level, but also at the local community, state, national and even international levels (e.g., natural disasters, pandemics, war) (29–34). One such event is the COVID-19 pandemic, a global event associated with the SARS-CoV-2 virus, resulting in numerous measures that directly and significantly impacted various aspects of human behavior. These include, but are not limited to, reduced freedom of movement, reduced access to essential needs, and restricted social interaction, purportedly directed to restrict the spread of a virus that was predicted to result in a significant excess in mortality (34–38). Such measures, impacting the very essence of humanity (39–42) and fundamental human behavior (e.g., social relationships, including within the family) also adversely and significantly impacted mental well-being (34, 42, 43).

The potential mental health impact of the COVID-19 pandemic is reflected in the significant increase, observed globally, in the volume of scientific literature addressing this subject, in various groups of the general population, including university students (44–54). Many factors associated with the COVID-19 pandemic, such as the fast spread of news on the internet, including social media, and its potential to induce fear (55), the presentation of the information related to the virus itself, the imposition of national and regional lockdowns, and the involvement of politics have been reported to produce detrimental effects, including in university-age students (42, 56–58). Such situations and conditions also have the potential to adversely impact other areas of life, including relationships (e.g., increased abuse within the family) (59–63) and the economy and finances (e.g., loss of job, loss of businesses) (64), which have also been shown to potentially contribute to decreased mental well-being (65, 66). In relation to university students specifically, this population also faced additional possible stressors related to education including, but not limited to, the transitioning of classes from in-person attendance to online formats and isolation from friends and peer support (36, 67–69). However, extensive research remains necessary to assist in providing a better comprehension of the magnitude of the impact of the COVID-19-related events on the emerging adult group and, more specifically, the university student population.

In times of stress, individuals often employ various coping mechanisms in order to counter or, at the very least, temper the impact of the stressors (70, 71). The stressor and the adopted coping mechanism ultimately impact psychological well-being. In fact, coping has been described as a mediator of emotion (72). Additionally, the relationship between the individual and their response (coping) and the environment from which the stress emanates is considered to be dynamic and bidirectional (17). Previous research further implicates coping as a mediator between mental health and a number of variables (e.g., resilience, psychological maltreatment, body image, locus of control, etc.) (73–78). Thus, the method of coping (e.g., emotion-focused or problem-focused), associated with how the stress is perceived, has the potential to influence the response, positively or negatively, with varying effects on an individual's mental health depending on the coping mechanisms utilized (74, 79–81).

While the impact of stress on mental health and the mediating role of coping mechanisms are substantially documented in the literature, they have not been addressed in regards to the COVID-19 pandemic and the university student population. Thus, given the above literature indicating that coping mechanisms can mediate mental health, and can potentially influence the impact of a stressful life event, in addition to the vulnerability of university students to negative mental health, the aim of this cross-sectional study was to test and verify the mediation of coping mechanisms (emotion-focused, problem-focused, dysfunctional coping) on the association between stress related to the ongoing COVID-19 pandemic and mental health in a sample of university students. It is proposed that the use of a cross-sectional study provides a unique point-in-time perspective of the impact of an event that could not be predicted (the pandemic). Additionally, the continued presence of an event has the potential to confound the measured parameters due to variable adaptation of the responses of the subjects being tested. Thus, according to the evidence provided in the above review, we proposed the following hypothesis: Coping strategies (emotion-focused, problem-focused, dysfunctional coping) have a mediating effect on the relationship between the impact of the COVID-19 pandemic (stressor) and mental health. It is expected that emotion-focused and problem-focused coping, considered to be positive coping strategies (82–86), would potentially aid in reducing the negative mental health impact, while dysfunctional coping would potentially have the opposite effect, that is, positively mediate (i.e., enhance) the negative impact of the event on mental health.

## Materials and Methods

### Study Design

In compliance with Federal Law, indicating that all researchers conducting testing on human participants must complete training on the protection of research subjects, all survey administrators completed the Protecting Human Research Participants Training Module provided by the NIH Office of Extramural Research. Certification is kept on file for documentation purposes. Prior to administration of the survey, Franciscan University of Steubenville Institutional Review Board (IRB) approval was obtained (#2020-08). Our cross-sectional study consisted of a convenience sample of university/college (undergraduate and graduate) students from Franciscan University of Steubenville, a small private Catholic university located in Steubenville, OH, United States. An anonymous survey was sent via the university student email address, to all students taking classes at Franciscan University, who were at least 18 years of age. Over the course of 4 weeks (August 31st to September 21st, 2020), the survey was administered through the online survey engine SurveyMonkey®. Prior to completing the survey, participants were directed to a consent form, which detailed the confidentiality and the nature of the study and explained that participation in the study implied consent to analyze and publish the overall results. Participants who did not provide consent were directed to the *Disqualification Page*. The projected time of completion of the survey was ~15–20 min. The instructions indicated to students that they should not spend too much time on any question and give their honest response. The final page of the survey included a link to enter an optional drawing for one (1) of 15 Amazon gift cards (5 of $20 and 10 of $10). The participants were informed that there was no possibility of linking the drawing information to that of the survey and that their information would remain confidential.

### Exclusion Criteria

Exclusion criteria included any individual who: (1) was younger than 18 years of age (*n* = 1), (2) was not a student at Franciscan University of Steubenville (*n* = 2) or did not complete the question (*n* = 2), (3) responded “No” (*n* = 5) or did not complete the question regarding consent (*n* = 26), (4) did not complete the survey question regarding their age (*n* = 10), (5) did not provide a response to the Depression, Anxiety and Stress Scale (*n* = 94) and (6) provided an ambiguous demographic response (*n* = 1). The final number of participants whose responses met inclusion criteria was 676 (out of the original 817 total respondents, i.e., 83%).

### Survey Structure

#### Demographic Questions

Demographic questions included: age, sex, class, number of semesters completed at Franciscan University, major, housing during the school year at the time of survey, and relationship status. Participants were also asked to indicate whether they were an online-only and/or a transfer student. If the participant indicated that they were a transfer student, they were asked to further indicate whether or not they transferred in the current semester.

#### Depression, Anxiety and Stress Scale (DASS-21)

The 21-question version of the DASS (87) was also included in the survey. This survey measures various core symptoms associated with depression (D; e.g., “*I felt down-hearted and blue*”), anxiety (A; e.g., “*I felt I was close to panic*”) and stress (S; e.g., “*I found it difficult to relax*”). Subjects were instructed to indicate how much each statement applied to them over the past week on a four-point Likert scale (from 0 = *Did not apply to me at all* to 3=*Applied to me very much, or most of the time*). The DASS-21 is not intended to diagnose disorders related to depression, anxiety or stress. The participants' total scores in the three criteria (D, A, and S) were categorized by severity as either “normal,” “mild,” “moderate,” “severe,” or “extremely severe,” as previously defined (88). The Cronbach's alpha for each of the three mental health parameters (depression, anxiety and stress) were α = 0.90, 0.82, and 0.86, respectively, indicating good to excellent internal consistency.

#### Questions on Pandemic-Related Sources of Stress and Perceived Effect of COVID-19 on Mental Health

Participants were asked to indicate the extent to which they believed their mental health changed relative to the time before the COVID-19 outbreak, as a result of the outbreak and its effects, on a five-point Likert scale (from *Much worse* to *Much better*). Additionally, participants were asked to indicate (on a five-point Likert scale from *Not at all* to *Extremely*) how significant a source of stress various items were in their lives relative to various factors associated with the COVID-19 pandemic, such as the possibility of contracting COVID-19, the closing of schools/universities, going to public places or a health care facility, etc.

#### Questions on Personal Experiences Surrounding COVID-19

Returning students (i.e., those who had previously attended Franciscan University) were asked questions regarding various aspects associated with the COVID-19 pandemic, including their level of satisfaction with the online classes at Franciscan University, their state of primary residence during the pandemic, and whether or not they returned home (i.e., place of primary residence during pandemic).

Freshmen, who would not have been attending a university during the initial outbreak of COVID-19, were asked equivalent questions in relation to their high school, as well as where they were living during the outbreak. Similarly, participants who transferred to Franciscan University the semester in which the survey was completed were asked similar questions in relation to the university from which they transferred (i.e., the university they were attending at the time of the COVID-19 outbreak).

#### Impact of Event Scale—Revised (IES-R)

The revised version of the Impact of Event Scale (89–91) was utilized to assess the participants' responses to a particular event (in this case, the COVID-19 pandemic). Individuals were asked to indicate on a five-point Likert scale (*Not at all* to *Extremely*) how distressing each item had been for them in the past seven days in relation to the COVID-19 pandemic. Total overall scores were calculated and categorized into the various levels of impact (89–91). Additionally, average scores were calculated for each of the following subscales: *Intrusion* (*INT*; e.g., “*I had dreams about it*”), *Avoidance* (*AVD*; e.g., “*I tried not to think about it*”), and *Hyperarousal* (*HYP*; e.g., “*I was jumpy and easily startled*”), for which reliability analysis indicated good internal consistency with Cronbach's alpha coefficients of α = 0.87, 0.85, and 0.81, respectively. The internal consistency for the *total* score, as calculated by Cronbach's alpha, showed a coefficient of α = 0.93.

#### Questions on COVID-19 Testing

In relation to COVID-19 testing, participants were asked to indicate whether or not they had been tested for COVID-19 prior to or since coming to campus (for Fall 2020 semester) and whether or not they tested positive. Moreover, questions were asked in relation to whether a close/immediate family member or a close friend had been tested for COVID-19 and whether or not they tested positive or negative.

#### Questions on Changes in Work Resulting From the COVID-19 Pandemic

Various survey questions were asked to assess potential changes in work that the participant, as well as family members and/or close friends, may have experienced as a result of the pandemic. Questions were adapted from the *Report on the Economic Well-Being of the U.S. Households in 2019* (64) and addressed whether or not the participant lost a job, experienced an increase or decrease in work hours (e.g., took paid leave, took unpaid leave, etc.) or applied for unemployment benefits since March 1, 2020 (the onset of the coronavirus outbreak in the United States). Equivalent questions were asked in regards to whether or not a close friend or a close/immediate family member had experienced the previously mentioned changes in work since March 1, 2020.

Additionally, participants were asked whether or not they were considered as a frontline/essential worker as defined by CISA (92) during the COVID-19 outbreak, as well as whether or not a close/immediate family member or a close friend fell under such a category.

#### Questions on Politics and Sources of News/Information

The survey also included questions assessing how much the participant depended on various sources of information pertaining to COVID-19 [e.g., CDC/WHO, Scientific sources (e.g., PubMed, PsycINFO, etc.), Social media, etc.] on a five-point Likert scale (*Not at all* to *Extremely*) and how often they watched/read various news sources on a six-point Likert scale (*Every day* to *Never*), as well as their rating of the trustworthiness of various news sources (e.g., CNN, Fox News, local newspaper, etc.) in regards to COVID-19 on a seven-point Likert scale (1 = *Very untrustworthy* to 7=*Very trustworthy*), with the possibility of indicating that they were not familiar with the news source. Questions were adapted from Dart et al. (93).

Additionally, a question was asked in regards to how the participant would describe themselves politically (*Extremely Conservative* to *Extremely Liberal*), as well as whether they think of themselves as Republican (*Strong Republican, Moderate Republican, Leaning Republican*), Democrat (*Strong Democrat, Moderate Democrat, Leaning Democrat*) or *Independent*.

#### Brief COPE

Our survey also included the Brief COPE (94), a shortened version of the original COPE, which assesses various coping mechanisms that individuals may utilize to cope with stress and deal with problems. Participants were instructed to rate each of the 28 items on a four-point Likert scale (from *I haven't been doing this at all* to *I've been doing this a lot*) relative to the COVID-19 pandemic. The 28 items of the Brief COPE are categorized into 14 different scales pertaining to different coping strategies (e.g., active coping, using emotional support, substance use, etc.) (94). Additionally, the 14 scales are then further categorized into *Problem-focused (PF*; e.g., “*I've been thinking hard about what steps to take*”*), Emotion-focused (EF*; e.g., “*I've been looking for something good in what is happening*”*)*, and *Dysfunctional coping (DC*; e.g., “*I've been giving up the attempt to cope*”*)* (95) strategies. Reliability analysis indicated good internal consistency, as measured by Cronbach's alpha, for PF and DC (α = 0.82 and 0.81, respectively) and acceptable internal consistency for EF (α = 0.78).

### Statistical Analysis

Analyses were conducted on all data remaining following the application of the exclusion criteria (*n* = 676) using R version 4.1.1, SigmaPlot version 14.0 (Systat Software, Inc.) and Jamovi version 2.2.2.0. Differences in proportions between the sexes for the DASS-21, IES-R, Brief COPE (14 subscales), perceived change in mental health and sources of stress relative to COVID-19, as well as within the sexes for perceived change in mental health were analyzed using Chi squared or Fisher's exact test, as appropriate. *T*-tests (two-tailed) were utilized in order to assess differences between sexes in the average scores of the DASS-21. Differences in the average IES-R subscale scores for those in a category of concern (i.e., score >23) were analyzed using a one-way repeated measures ANOVA, whereas the average Brief COPE subscale scores (three subscales) were analyzed using a two-way repeated measures ANOVA with one factor repetition (Brief COPE subscales) due to the inclusion of sex as a variable in the analysis. Two-way repeated measures ANOVA with one-factor repetition (Brief COPE subscale or EFA factor) was also used to assess differences in the average Brief COPE scores across subscales or average score of the EFA-identified factors between participants scoring below (*Normal*) or within a level of concern for PTSD (*PTSD*). Tukey *post-hoc* analysis was conducted where appropriate. In order to investigate the patterns within the various COVID-19-related sources of stress and uncover specific factors, exploratory factor analysis (EFA) was utilized in this study to explore such patterns rather than to confirm a specific hypothesis relating to the various variables presented to the participants. Bartlett's test of sphericity and the Kaiser-Meyer-Olkin (KMO) measure of sampling adequacy were utilized to determine the factorability of the data pertaining to the COVID-19-related sources of stress. Pearson correlations were utilized to assess the relationship between the DASS-21, IES-R, Brief COPE and perceived change in mental health. Given the correlations between the IES-R and DASS-21 subscales and previous literature indicating a relationship between stressful events and mental health, in addition to the reported mediation of such a relationship by various coping behaviors (see **Figure 3A**) (72), mediation analysis using Baron and Kenny's criteria (96) and using 1,000 bootstrapping replicates (97) was utilized to investigate the potential relationship between the impact of the COVID-19 pandemic (predictor) on the mental health of the participants (outcome) and the potential mediating effect of coping strategies (mediator) on such a relationship.

## Results

### Current Demographics

The distribution of participants was 31% male and 69% female, which is relatively representative of the student population at Franciscan University of Steubenville. The demographic information pertaining to age, class, living status, relationship status, number of semesters completed at Franciscan University, online-only, transfer status, political standing and political party affiliation are presented in Table 1. Additionally, information pertaining to the frequency that participants accessed various sources of information for news and the perceived trustworthiness of various news sources are shown in Supplementary Figure 1 and Figure 2, respectively. This information represents the students during the semester in which the survey was conducted.

**Table 1:** Summary of current demographic variables.

**Variable**	**Male (M) *n* (%)**	**Female (F) *n* (%)**
**Age**		
18	40 (19.1)	96 (20.6)
19	31 (14.8)	75 (16.1)
20	44 (21.1)	101 (21.6)
21	27 (12.9)	68 (14.6)
22	17 (8.1)	29 (6.2)
23+	50 (23.9)	98 (21.0)
**Class**		
Freshman	50 (25.5)	95 (21.8)
Sophomore	34 (17.3)	83 (19.1)
Junior	37 (18.9)	95 (21.8)
Senior	38 (19.4)	76 (17.5)
Graduate	37 (18.9)	86 (19.8)
**Online-only?**		
Yes	33 (15.8)	92 (19.7)
No	176 (84.2)	375 (80.3)
**Transfer?**		
Yes	23 (15.8)	65 (19.1)
No	123 (84.2)	275 (80.9)
**If yes, transfer this semester?**		
Yes	10 (43.5)	24 (36.9)
No	13 (56.5)	41 (63.1)
**Semesters completed**		
<1	68 (32.5)	138 (29.6)
1–2	45 (21.5)	109 (23.3)
3–4	52 (24.9)	119 (25.5)
5–6	31 (14.8)	65 (13.9)
7–8	10 (4.8)	22 (4.7)
9+	3 (1.4)	14 (3.0)
**Living status**		
Main campus—M/F only Dorms	77 (36.8)	199 (42.6)
Main campus—Co-ed Dorms	15 (7.2)	39 (8.4)
Main campus—Assisi Heights	16 (7.7)	53 (11.3)
Lower campus	21 (10.0)	20 (4.3)
Off campus	80 (38.3)	156 (33.4)
**Share room**		
Yes	153 (73.2)	336 (71.9)
No	56 (26.8)	131 (28.1)
**Relationship status**		
Single	121 (57.9)	290 (62.1)
In a relationship	49 (23.4)	98 (21.0)
Married	27 (12.9)	47 (10.1)
Discerning religious life/priesthood	11 (5.3)	16 (3.4)
Priest or other religious	1 (0.5)	7 (1.5)
Divorced/Separated	0 (0.0)	6 (1.3)
Widow(er)	0 (0.0)	3 (0.6)
**Political standing**		
Conservative	149 (81.4)	338 (83.5)
Moderate	22 (12.0)	43 (10.6)
Liberal	12 (6.6)	24 (5.9)
**Political party affiliation**		
Republican	120 (65.6)	303 (74.8)
Independent	56 (30.6)	90 (22.2)
Democrat	7 (3.8)	12 (3.0)

*Age, Online-only status, Semesters completed, Living status and Relationship status—Male (M): n = 209, Female (F): n = 467; Class—M: n = 196, F: n = 435; Transfer status—M: n = 146, F: n = 340; Transfer this semester—M: n = 23, F: n = 65; Satisfaction with online classes—M: n = 181, F: n = 408; Political standing and Political party affiliation—M: n = 183, F: n = 405. Assisi Heights: On-campus apartments available to juniors and seniors; Co-ed dorms: M/F dorms with co-ed common areas; Lower Campus: motel-style dorms located on the periphery of the main campus*.

### Pandemic-Related Demographics

**Figure 1:**
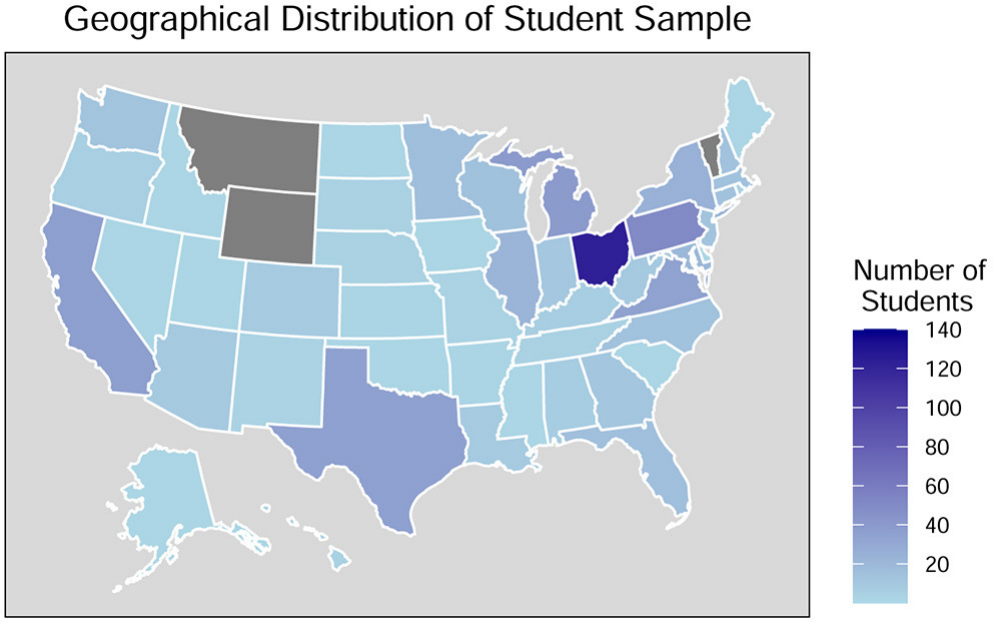
Geographical distribution of student sample during the COVID-19 outbreak (*n* = 617). Data excludes those who were not residing in the United States (*n* = 7). Gray-colored states reflect no representation in sample.

The geographical distribution of the student sample (*N* = 617), excluding those who were not residing in the United States (*n* = 7) and those who provided ambiguous responses (*n* = 2) is shown in Figure 1. Additional demographics pertaining specifically to the outbreak of the pandemic, including place of residence, satisfaction with online classes, COVID-19 testing, work, frontline worker status and sources of information pertaining to COVID-19, are included in Table 2.

**Table 2:** Summary of pandemic-related demographic variables.

**Variable**	***n*** **(%)**
**Residing at home during outbreak/pandemic**	
Freshmen (*N* = 145)	141 (97.2)
Transfer students (*N* = 24)	24 (100)
Returning students (*N* = 434)	421 (97.0)
**Satisfaction with online classes during pandemic (*****N*** **=** **589)**	
Unsatisfied	210 (35.7)
Neutral	76 (12.9)
Satisfied	303 (51.4)
**COVID-19 testing**	
**1. Self**	
• Tested prior to coming to campus (*N* = 608)	87 (14.3)
• Tested positive	5 (5.7)
• Tested since coming to campus (*N* = 607)	61 (10.0)
• Tested positive	6 (9.8)
**2. Close/immediate family member (*****N*** **=** **606)**	
• Tested but negative	301 (49.7)
• Tested positive	69 (11.4)
**3. Close friend (*****N*** **=** **606)**	
• Tested but negative	314 (51.8)
• Tested positive	150 (24.8)
**Work during pandemic**	
**1. Self (*****N*** **=** **596; 3 data points removed due to ambiguous responses)**	
-I did not have a job	165 (27.7)
-Lost a job, got laid off, or told not to work any hours (on-campus/SWOP)	87 (14.6)
-Lost a job, got laid off, or told not to work any hours (off-campus)	88 (14.8)
-Voluntarily quit or changed jobs	78 (13.1)
-Reduced hours or took unpaid leave	67 (11.2)
-Took paid leave (including sick or vacation time)	17 (2.9)
-Increased hours worked or worked overtime	137 (23.0)
-Applied for unemployment benefits	44 (7.4)
-None of the above	127 (21.3)
**2. Close/immediate family member (*****N*** **=** **599)**	
-Lost a job, got laid off, or told not to work any hours	238 (39.7)
-Voluntarily quit or changed jobs	111 (18.5)
-Reduced hours or took unpaid leave	215 (35.9)
-Took paid leave (including sick or vacation time)	180 (30.1)
-Increased hours worked or worked overtime	243 (40.6)
-Applied for unemployment benefits	194 (32.4)
**3. Close friend (*****N*** **=** **599)**	
-Lost a job, got laid off, or told not to work any hours	339 (56.6)
-Voluntarily quit or changed jobs	191 (31.9)
-Reduced hours or took unpaid leave	261 (43.6)
-Took paid leave (including sick or vacation time)	170 (28.4)
-Increased hours worked or worked overtime	250 (41.7)
-Applied for unemployment benefits	287 (47.9)
**Frontline worker in pandemic (*****N*** **=** **599)**	
Self	193 (32.2)
Close/immediate family member	359 (59.9)
Close friend	292 (48.7)
**Dependency on various sources for information pertaining to COVID-19 (*****N*** **=** **599)**	
-Family	275 (46.3)
-Medical Professionals	211 (35.5)
-Friends	172 (29.0)
-CDC/WHO	142 (23.9)
-News sources—online or print (e.g., FoxNews, CNN, local newspapers, etc.)	138 (23.2)
-Scientific sources (e.g., PubMed, PsycINFO, etc.)	93 (15.7)
-Teachers/Professors	88 (14.8)
-Social Media	83 (14.0)
-Medical pamphlets/websites (e.g., Mayo Clinic website)	79 (13.3)
-Other	46 (7.7)

*Percentages shown will not add up to 100% (with the exception of the question pertaining to Satisfaction with online classes during pandemic) as they reflect those who responded “Yes” or “True” and are out of the total number responding to the specific questions or subcategory; Dependency on various sources for information pertaining to COVID-19—the percentages are those who responded depending on the specific source of information Moderately or Extremely out of the total number responding to this specific question*.

### Sources of Stress Relative to COVID-19

The percentage of students (total, males and females) who reported that various potential sources of stress related to the COVID-19 pandemic were a moderate or extreme source of stress are shown in Table 3.

**Table 3:** COVID-19 sources of stress.

**Factor**	**Source of stress**	**Total (%)**	**Male (*n* = 197; %)**	**Female (*n* = 435; %)**	**χ^2^ (1, *N* = 632)**
IntF	Not being able to attend church service/Mass	71.0	59.9	76.1	16.51^***^
IntF	Social aspect/not being able to get together with people	56.0	40.6	63.0	26.66^***^
IntF	Possibility of life not going back to the way it was before	50.5	36.0	57.0	23.02^***^
IntF	Possibility of losing freedoms	50.2	42.6	53.6	6.04^*^
EdRout	Closing of schools/universities	49.4	37.6	54.7	15.28^***^
IntF	Possibility of government overreach	47.5	47.7	47.4	3.68
EdRout	Change in modality of teaching (e.g., from on ground to online classes)	43.7	35.0	47.6	8.19^**^
TrnsV	Possibility of interruption of my life because of testing positive	42.4	30.5	47.8	16.03^***^
TrnsV	Possibility of interruption of my life because of someone close testing positive	36.7	24.4	42.3	18.01^***^
EdRout	Being at home	35.4	23.9	40.7	16.06^***^
TrnsV/AnxApp	Possibility of a close/immediate family member contracting COVID-19	33.4	23.4	37.9	12.32^***^
TrnsV	Possibility of passing the virus on to someone, including as an asymptomatic carrier	32.6	13.7	41.1	45.24^***^
CovMed	News/media reports relative to COVID-19	27.5	18.8	31.5	10.36^**^
CovMed	Social media relative to COVID-19	26.9	20.3	29.9	5.85^*^
ChngLife	Change in exercise habits	23.6	14.2	27.8	13.18^***^
AnxApp	Going to public places (e.g., grocery stores, parks, church, etc.)	21.7	12.7	25.7	12.86^***^
AnxApp	Possibility of shortages in food and other essential supplies	21.0	11.7	25.3	14.31^***^
ChngLife	Change in eating habits	20.3	10.2	24.8	17.18^***^
TrnsV / AnxApp	Possibility of a close friend contracting COVID-19	19.8	10.7	23.9	14.18^***^
	Possibility of government inaction	19.1	13.2	21.8	5.99^*^
ChngLife	Change in sleep habits	17.4	8.6	21.4	14.46^***^
AnxApp	Going to a health care facility (e.g., doctor/dentist, etc.)	15.7	8.6	18.9	9.96^**^
TrnsV / AnxApp	Possibility of contracting COVID-19	14.9	9.1	17.5	6.79^**^
AnxApp	Eating food prepared by others	6.3	5.1	6.9	0.48

*Data reflects the percentage of participants (across sex and combined) reporting Moderately or Extremely for how significant a source of stress, relating to the COVID-19 outbreak, they perceived each item to be in their lives. Colors represent the different EFA-identified factors. IntF, Interference with Freedom; EdRout, Education Routine; TrnsV, Transmission of COVID-19 Virus; CovMed, COVID-19-Related Media; ChngLife, Changes in Lifestyle Factors; Anx/App, Anxiety/Apprehension. Possibility of a close/immediate family member contracting COVID-19, Possibility of a close friend contracting COVID-19, Possibility of contracting COVID-19 are included in two EFA factors. Proportions tests were conducted on the differences between males and females for each source of stress*.

* *p < 0.05*,

** *p < 0.01*,

*** *p < 0.001*.

#### Exploratory Factor Analysis for the COVID-19-Related Sources of Stress

Exploratory factor analysis (EFA) using a principal-axis factor extraction (98, 99) was used to investigate the potential factors present within the sources of stress related to COVID-19 included in our survey. Parallel analysis (98, 99) recommended a six-factor solution (see Table 4). Given the high correlation of the items, a “promax” (oblique) rotation (98, 99) was utilized for interpretation of the six factors. This rotation had sums of squared loadings ranging from 1.67 to 2.90. The correlation coefficients between factors ranged from 0.168 to 0.665.

**Table 4:** Summary of exploratory factor analysis results pertaining to the items addressing COVID-19-related sources of stress, using the principal axis factoring extraction method in combination with a promax rotation (*n* = 632).

**Factor loadings**							
	**Factor**						
	**Interference with freedom**	**Transmission of COVID-19 virus**	**Anxiety/ apprehension**	**Changes in lifestyle factors**	**Education routine**	**COVID-19-related media**	**Uniqueness**
Contract COVID-19—self		0.545	0.353				0.392
Contract COVID-19—family		0.616	0.333				0.400
Contract COVID-19—friend		0.621	0.389				0.333
Pass to Other		0.589					0.495
Interruption—self positive		0.785					0.387
Interruption—other positive		0.813					0.347
News						0.908	0.168
Social media						0.923	0.179
Teaching change					0.826		0.401
Close schools					0.905		0.206
Home					0.497		0.543
Lose freedoms	0.906						0.279
Govt overreach	0.909						0.414
Govt inaction							0.735
Life different	0.651						0.428
Social aspect	0.455						0.471
No church	0.452						0.629
Supply shortage			0.534				0.587
Public places			0.869				0.363
Health care facility			0.758				0.387
Food by others			0.560				0.571
Sleep change				0.708			0.371
Exercise change				0.866			0.331
Eating change				0.887			0.194

*Contract COVID-19 - Self, Possibility of contracting COVID-19; Contract COVID-19 - Family, Possibility of a close/immediate family member contracting COVID-19; Contract COVID-19—Friend, Possibility of a close friend contracting COVID-19; Pass to Other, Possibility of passing the virus on to someone, including as an asymptomatic carrier; Interruption—Self Positive, Possibility of interruption of my life because of testing positive; Interruption—Other Positive, Possibility of interruption of my life because of someone close testing positive; News, News/media reports relative to COVID-19; Social Media, Social media relative to COVID-19; Teaching Change, Change in modality of teaching (e.g., from on ground to online classes); Close Schools, Closing of schools/universities; Home, Being at home; Lose Freedoms, Possibility of losing freedoms; Govt Overreach, Possibility of government overreach; Govt Inaction, Possibility of government inaction; Life Different, Possibility of life not going back to the way it was before; Social Aspect, Social aspect/not being able to get together with people; No Church, Not being able to attend church services/Mass; Supply Shortage, Possibility of shortages in food and other essential supplies; Public Places, Going to public places (e.g., grocery stores, parks, church, etc.); Health Care Facility, Going to a health care facility (e.g., doctor/dentist, etc.); Food by Others, Eating food prepared by others; Sleep Change, Change in sleep habits; Exercise Change, Change in exercise habits; Eating Change, Change in eating habits*.

The first factor, identified as “Interference with Freedom” (*IntF*), included the possibility of losing freedoms (Lose Freedoms), the possibility of government overreach (Govt Overreach), the possibility of life not going back to the way it was before (Life Different), the social aspect and not being able to get together with people (Social Aspect), and not being able to attend church services/Mass (No Church). The second factor, labeled as “Transmission of COVID-19 Virus” (*TrnsV*), included the possibility of self (Contract COVID-19—Self), a close/immediate family member (Contract COVID-19—Family) or a close friend (Contract COVID-19—Friend) contracting COVID-19, the possibility of passing the virus on to someone, including as an asymptomatic carrier (Pass to Other), and the possibility of interruption of life due to self (Interruption—Self Positive) or someone close (Interruption—Other Positive) testing positive. The third factor, “Anxiety/Apprehension” (*AnxApp*), also included the possibility of self, a close/immediate family member or a close friend contracting COVID-19, as well as the possibility of shortages in food and other essential supplies (Supply Shortage), going to public places (e.g., grocery stores, parks, church, etc.) (Public Places), going to a health care facility (e.g., doctor/dentist, etc.) (Health Care Facility), and eating food prepared by others (Food by Others). The fourth factor, identified as “Changes in Lifestyle Factors” (*ChngLife*), included changes in sleep (Sleep Change), exercise (Exercise Change) and eating (Eating Change) habits, all factors involved in the overall health and well-being of the individual. The fifth factor, labeled as “Education Routine” (*EdRout*), included change in modality of teaching (e.g., from on ground to online classes) (Teaching Change), the closing of schools/universities (Close Schools) and being at home (Home). The final factor, “COVID-19-Related Media” (*CovMed*), included news/media reports (News) and social media (Social Media) relative to COVID-19. Of note, the possibility of government inaction (Govt Inaction) as a potential source of stress was not included in any factor following EFA.

### Mental Health

#### DASS-21

Based on the scoring of the DASS-21 as previously described (87), from the participants who completed this section of the survey (*N* = 676), the overall percentages for each of the categories was as follows: Normal: 59.6, 62.1, and 67.5%; Mild: 12.1, 7.8, 9.2%; Moderate: 15.4, 14.5, 12.0%; Severe: 5.9, 6.5, 8.7%; Extremely Severe: 7.0, 9.0, 2.7% for depression, anxiety and stress, respectively. In relation to the average scores for each of the subscales, analysis revealed significantly higher average anxiety [*t*_(674)_ = 4.894, *p* < 0.001, *r*^2^ = 0.034] and stress [*t*_(674)_ = 5.124, *p* < 0.001, *r*^2^ = 0.037] scores in females relative to males, as well as a tendency toward significance [*t*_(674)_ = 1.711, *p* = 0.088, *r*^2^ = 0.004] in average depression scores with females scoring higher than males. Additionally, the sex differences in relation to the proportions pertaining to the combined severe and extremely severe categories for each of the subscales are provided in Table 5.

**Table 5:** Percentages and proportions test outputs between the sexes pertaining to the DASS-21, IES-R and Brief COPE survey scales.

**Variable**	**Male (M) *n* (%)**	**Female (F) *n* (%)**	**χ^2^ [1, ∑n(M,F)]**
**DASS-21 (severe and extremely severe)**			
Male: *n* = 209; Female: *n* = 467			
Depression	20 (9.6)	67 (14.3)	2.53
Anxiety	15 (7.2)	90 (19.3)	15.19^***^
Stress	14 (6.7)	63 (13.5)	5.94^*^
**IES-R**			
Male: *n* = 192; Female: *n* = 416			
24–32	10 (5.2)	56 (13.5)	8.41^**^
33–36	2 (1.0)	18 (4.3)	3.48^†^
37+	11 (5.7)	62 (14.9)	9.62^**^
**Brief COPE (Score** **≥5)**			
Male: *n* = 180; Female: *n* = 402			
*Problem-focused*			
Active coping	24 (13.3)	56 (13.9)	0.00
Planning	20 (11.1)	50 (12.4)	0.10
Using instrumental support	16 (8.9)	44 (10.9)	0.37
*Emotion-focused*			
Acceptance	76 (42.2)	141 (35.1)	2.42
Humor	57 (31.7)	79 (19.7)	9.36^**^
Religion	68 (37.8)	78 (44.3)	1.90
Using emotional support	16 (8.9)	52 (12.9)	1.60
Positive reframing	30 (16.7)	99 (24.6)	4.12^*^
*Dysfunctional coping*			
Self-distraction	30 (16.7)	112 (27.9)	7.85^**^
Denial	3 (1.7)	6 (1.5)	–
Venting	11 (6.1)	22 (5.5)	0.01
Substance use	4 (2.2)	7 (1.7)	–
Behavioral disengagement	6 (3.3)	16 (4.0)	0.02
Self-blame	8 (4.4)	27 (6.7)	0.77

*Missing **χ**^2^ are due to Fisher's exact test being conducted. ∑n(M,F): Sum of male and female participants shown under the “Variable” column*.

* *p < 0.05*,

** *p < 0.01*,

*** *p < 0.001*.

† *< 0.05 < p < 0.1*.

#### Perceived Change in Mental Health as a Result of COVID-19

Analysis of the question assessing the extent that the participants believed their mental health changed relative to the time before the COVID-19 outbreak indicated a significantly higher proportion [χ^2^ (1, *N* = 632) = 9.30, *p* < 0.01] of females (53.6%) relative to males (40.1%) reporting *worse* (*Somewhat worse* or *Much worse*) mental health. Moreover, a significantly higher proportion of males relative to females reported perceiving their mental health to have remained *About the same* [χ^2^ (1, *N* = 632) = 7.03, *p* < 0.01; Males: 45.2%, Females: 33.8%]. Additionally, there was no significant difference between the proportion of males and females who reported that they believed their mental health was *better* (*Somewhat better* or *Much better*) [χ^2^ (1, *N* = 632) = 0.34, *p* > 0.05; Males: 14.7%, Females: 12.6%].

Within the specific sexes, the greatest proportion of females reported *worse* followed by *About the same* and *better* mental health. All comparisons were significant (all *p* < 0.001) [χ^2^ (2, *N* = 435) = 163.94, *p* < 0.001]. Relative to males, a significantly higher proportion [χ^2^ (2, *N* = 197) = 47.21, *p* < 0.001] reported *worse* and *About the same* relative to *better* (both *p* < 0.001). However, unlike females, there was no significant difference between the proportion of males who reported *worse* and *About the same* (*p* > 0.05).

### IES-R

A significantly [χ^2^ (1, *N* = 608) = 28.12, *p* < 0.001] higher proportion of females (32.7%) relative to males (12.0%) reported total IES-R scores within the published cutoff values for PTSD (100–102). The sex differences in relation to the proportions pertaining to each of the severity levels of PTSD symptoms (*24–32, 33–36, 37*+) are provided in Table 5. Moreover, within each category of severity of the IES-R total score, the average scores reported were not significantly different between the sexes (*24–32*—Females: M = 28.09, SEM = 0.34; Males: M = 27.40, SEM = 0.72; *33–36*—Females: M = 34.39, SEM = 0.23; Males: M = 34.50, SEM = 0.50; and *37*+—Females: M = 46.84, SEM = 1.13; Males: M = 45.73, SEM = 2.55; all *p* > 0.05).

The IES-R scores were sub-categorized into three subscales (intrusion, INT; avoidance, AVD; hyperarousal, HYP) according to Weiss (91). Given that the average INT, AVD and HYP scores were not significantly different between the sexes (all *p* > 0.05), the data for both sexes was combined and analyzed to assess the differences between the average scores for each subscale. This analysis indicated a significant difference between subscale scores, *F*_(2,316)_ = 27.25, *p* < 0.001, η^2^ = 0.076. *Post-hoc* analysis indicated that the average AVD score (M = 1.97, SEM = 0.05) was significantly higher than both INT and HYP average scores (INT: M = 1.55, SEM = 0.05; HYP: M = 1.55, SEM = 0.06; both *p* < 0.001). There was no significant difference between the INT and HYP average scores (*p* > 0.05).

### Brief COPE

In relation to coping strategies, as measured by the Brief COPE, the results indicated a significant effect of sex [*F*_(1,1160)_ = 9.77, *p* < 0.01, η^2^= 0.008) and the subscales (emotion-focused, EF; problem-focused, PF; dysfunctional coping, DC; *F*_(2,1160)_ = 451.28, *p* < 0.001, η^2^ = 0.215). However, there was no significant effect of the interaction between sex and Brief COPE subscales [*F*_(2,1160)_ = 0.00, *p* > 0.05, η^2^ = 0.000). *Post-hoc* analysis indicated that the average EF score (Females: M = 1.60, SEM = 0.03; Males: M = 1.46, SEM = 0.04) was significantly higher than both PF (Females: M = 1.17, SEM = 0.03; Males: M = 1.04, SEM = 0.05) and DC (Females: M = 0.73, SEM = 0.02; Males: M = 0.60, SEM = 0.03) scores for both males and females. Additionally, PF was significantly higher than DC for both sexes (all *p* < 0.001). Moreover, females scored significantly higher than males in all three subscales (all *p* < 0.05).

The three subscales of the Brief COPE are further broken down into 14 subcategories addressing the frequency of utilization of specific coping mechanisms. The breakdown of the proportions for the sexes reporting a score ≥5 (at least one score indicating “*I've been doing this a lot*” and one score of “*I've been doing this a medium amount*,” or both scores indicating “*I've been doing this a lot*”) for each of the 14 categories of coping strategies, measured by the Brief COPE, are shown in Table 5.

In addition to the above relationships, we were also interested in how our results compared to the breakdown provided by Mohr, Delfino et al. (103, 104) addressing *Activity* (AC, *activity-passivity*) and *Defeatism* (DF, *defeatism-resilience*). Using the criteria addressed by Delfino et al. (104) defining “high defeatism” as being ≥1.3 standard deviations above the mean and reflecting insufficient coping behavior under chronic stress, our results indicate that 10.5% of the sample students met this criterion.

### Correlation of DASS-21, IES-R, Brief COPE, and Perception of Mental Health Change Relative to COVID-19 Outbreak

**Figure 2:**
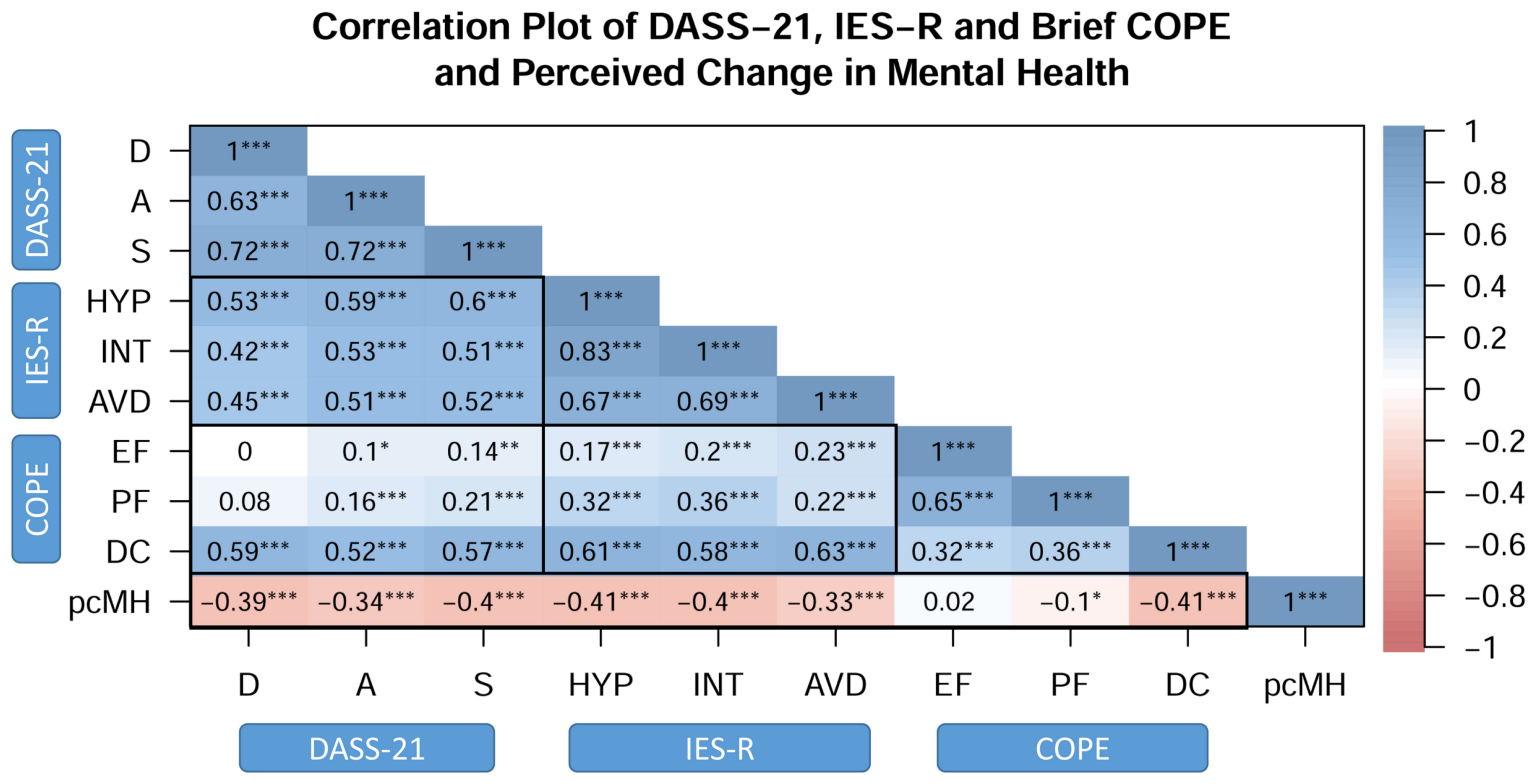
Correlation plot of mental health outcomes (DASS-21), the impact of the COVID-19 pandemic (IES-R), coping strategies (Brief COPE) and perceived change in mental health. ***DASS-21***: D, depression; A, anxiety; S, stress; ***IES-R***: HYP, hyperarousal; INT, intrusion; AVD, avoidance; ***COPE***: Brief COPE, EF, emotion-focused coping; PF, problem-focused coping; DC, dysfunctional coping; ***pcMH***: perceived change in mental health relative to before the COVID-19 outbreak. *p < 0.05, **p < 0.01, ***p < 0.001.

Correlation analysis indicated various significant positive relationships (Figure 2) between the variables measured pertaining to mental health (DASS-21; D, A, S), the impact of the COVID-19 pandemic on the participant (IES-R; HYP, INT, AVD) and coping mechanisms (Brief COPE; EF, PF, DC). Moreover, various significant negative correlations were revealed between these measures and the question regarding the participant's perceived change in mental health relative to before the COVID-19 outbreak. Given the coding of the question pertaining to mental health change relative to the COVID-19 outbreak, with “much worse” coded as “0” to “much better” coded as “4,” a negative correlation indicates that the higher the score on the various scales (DASS-21, IES-R and Brief COPE), the worse (lower score) the participant perceived their mental health to be.

In relation to the breakdown of the Brief COPE provided by Mohr et al., and addressed above, our results indicated strong and significant relationships between *dysfunctional coping* and *defeatism* (*r* = 0.89, *p* < 0.001), as well as between both *problem-focused* and *emotion-focused* coping and *activity* (PF vs. AC: *r* = 0.88; EF vs. AC: *r* = 0.87; both *p* < 0.001). The relationships between DF and the mental health measures (DF vs. D: *r* = 0.50, A: *r* = 0.49 and S: *r* = 0.49; all *p* < 0.001) are similar to those reported for DC and the mental health measures.

### Mediation Analysis of DASS-21, IES-R, and Brief COPE

**Figure 3:**
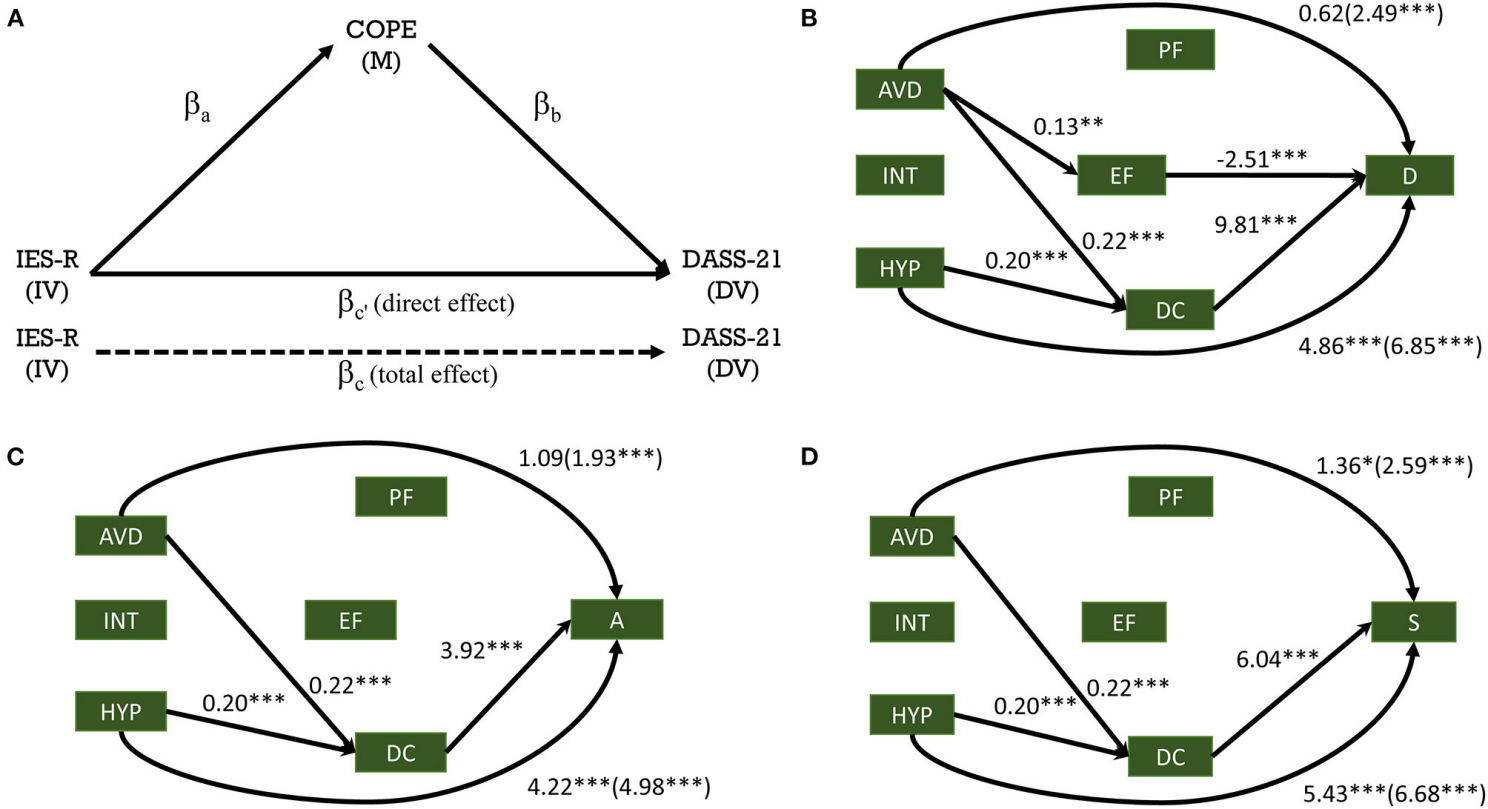
Mediation analysis of the impact of the COVID-19 pandemic on mental health, mediated through coping strategies. Hypothesized mediation model **(A)**; Final mediation model for depression **(B)**, anxiety **(C)** and stress **(D)**. ***DASS-21***: D, depression; A, anxiety; S, stress; ***IES-R***: HYP, hyperarousal; INT, intrusion; AVD, avoidance; ***COPE***: Brief COPE, EF, emotion-focused coping; PF, problem-focused coping; DC, dysfunctional coping. Data represents estimates/betas—direct (total). IV, independent variable; DV, dependent variable; M, mediator. *p < 0.05, **p < 0.01, ***p < 0.001.

Mediation analysis was performed to assess the mediating role of coping strategies (M = Mediator; PF, EF, and DC) on the relationship between the stressful responses caused by the impact of an event (IV = independent variable; INT, AVD and HYP) and mental health (DV= dependent variable; D, A, and S). Figure 3 shows the hypothesized conceptual model (Figure 3A) along with the final models (Figures 3B–D) for each of the mental health parameters (D, A, and S, respectively). The significant mediation pathways, including the indirect relationships, addressed in Table 6 (full results shown in Supplementary Tables 1–3), revealed that the total effect of AVD, INT and HYP on Depression scores were significant (H1: β = 2.49, *t* = 4.43, *p* < 0.001; β = −2.11, *t* = −2.39, *p* < 0.05; β = 6.85, *t* = 8.14, *p* < 0.001, respectively). Moreover, both Anxiety and Stress revealed similar behaviors. The results indicated a significant total effect for both Anxiety and Stress in relation to AVD (H1: β = 1.93, *t* = 4.32, *p* < 0.001; β = 2.59, *t* = 5.00, *p* < 0.001, for Anxiety and Stress, respectively) and HYP (H1: β = 4.98, *t* =7.46, *p* < 0.001; β = 6.68, *t* =8.63, *p* < 0.001 for Anxiety and Stress, respectively). The total effect relating to INT was not significant in either Anxiety or Stress (H1: β = 0.17, *t* = 0.25, and β = −1.06, *t* = −1.30, respectively, both *p* > 0.05).

**Table 6:** Summarized significant mediation pathways including the indirect relationships.

**Total effect** **(IV → DV)—c**		**Direct effect** **(IV → DV)—c'**		Indirect effects of IV on DV (a*b)					
**Coefficient**	***p*** **-value**	**Coefficient**	***p*** **-value**		**Coefficient**	**SE**	***T*** **-value**	***P*** **-values**	**95% CI**
2.493	<0.001	0.623	>0.05	AVD → EF → D	−0.325	0.145	−2.241	0.025	−0.638; −0.077
				AVD → DC → D	2.139	0.338	6.330	<0.001	1.498;2.817
6.847	<0.001	4.863	<0.001	HYP → DC → D	2.011	0.516	3.899	<0.001	1.097;3.120
1.927	<0.001	1.092	>0.05	AVD → DC → A	0.854	0.233	3.666	<0.001	0.404;1.327
4.977	<0.001	4.224	<0.001	HYP → DC → A	0.803	0.280	2.868	0.004	0.348;1.484
2.586	<0.001	1.356	<0.05	AVD → DC → S	1.317	0.271	4.862	<0.001	0.797;1.867
6.684	<0.001	5.430	<0.001	HYP → DC → S	1.238	0.371	3.333	<0.001	0.588;2.062

*Full results are available in Supplementary Tables 1–3. IV, Independent variable; DV, Dependent variable; AVD, Avoidance; HYP, Hyperarousal; EF, Emotion-Focused coping; DC, Dysfunctional Coping; D, Depression; A, Anxiety; S, Stress*.

With the inclusion of the mediating variables (PF, EF, DC), the impact of the independent variable (impact of event) was affected differentially between the three mental health outcomes (D, A, S). Specifically, the impact of HYP on D, A, and S was still found significant (β = 4.86, *t* = 5.03; β = 4.22, *t* = 4.24; β = 5.43, *t* = 6.15, respectively; all *p* < 0.001). However, the impact of INT and AVD on the depression scores (D), showed a tendency toward significance (INT: β = −1.87, *t* = −1.93, *p* = 0.054) or did not remain significant (AVD: β =0.62, *t* = 0.99, *p* > 0.05). In relation to A and S, the impact of INT and AVD was not significant (INT: β = 0.28, *t* = 0.32, *p* > 0.05; β = −1.18, *t* = −1.33, *p* > 0.05 for A and S, respectively) or showed a tendency toward significance for A in relation to AVD (β = 1.09, *t* = 1.86, *p* = 0.063). In relation to S, the impact of AVD was reduced but remained significant, β = 1.36, *t* = 2.04, *p* < 0.05.

The indirect effect of the impact of event as measured by HYP on Depression, Anxiety and Stress scores through DC was found significant (β = 2.01, *t* = 3.90, *p* < 0.001; β = 0.80, *t* = 2.87, *p* < 0.01; β = 1.24, *t* = 3.33, *p* < 0.001 for D, A, and S, respectively). In a similar fashion, the impact of event as measured by AVD on Depression, Anxiety and Stress scores through DC was found significant (β = 2.14, *t* = 6.33; β = 0.85, *t* = 3.67; β = 1.32, *t* = 4.86, all *p* < 0.001 for D, A, and S, respectively). The indirect effect of AVD on mental health through EF was only significant in the case of Depression (β = −0.33, *t* = −2.24, *p* < 0.05).

Thus, our findings indicate that the relationship between the impact of the event being addressed in this study (“with respect to the Coronavirus Disease 2019 (COVID-19) pandemic, which occurred starting around December 2019”) and mental health (D, A, and S) was primarily mediated by *dysfunctional coping* (DC) for all mental health measures, while depression was also mediated by *emotion-focused* (EF) coping.

### Brief COPE and COVID-19-Related Sources of Stress Across IES-R Outcome

**Figure 4:**
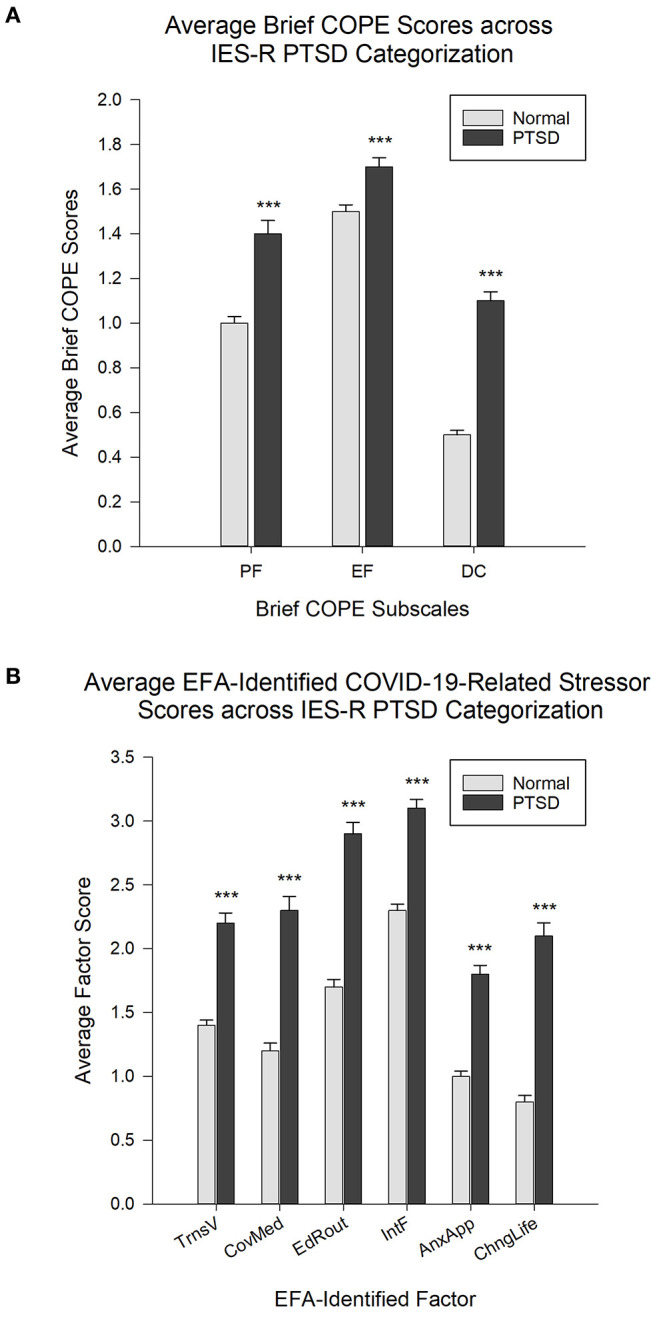
**(A)** Average Brief COPE scores for each subscale across IES-R categorization of scores falling within the normal range (<24, Normal) and those falling within the range of clinical concern for PTSD (≥24, PTSD). EF, emotion-focused coping; PF, problem-focused coping; DC, dysfunctional coping. *N* = 582; *Normal*: *n* = 426; *PTSD*: *n* =156. **(B)** Average factor scores, with higher scores interpreted as higher likelihood of perceiving the stressors as more stressful, across IES-R categorization of *Normal* (<24, Normal) and of clinical concern for *PTSD* (≥24, PTSD). *N* = 608; *Normal*: *n* = 449; *PTSD*: *n* = 159. ***p < 0.001 relative to the *Normal* group. Data is expressed as Mean ± SEM.

Pertaining to the Brief COPE subscales, when the IES-R scores were categorized as normal (<24, *Normal*) or of clinical concern for PTSD (≥24, *PTSD*), analysis indicated a significant difference across IES-R category [*Normal* vs. *PTSD*, *F*_(1,1160)_ = 98.13, η^2^ = 0.068], coping strategy [EF, PF and DC, *F*_(2,1160)_ = 343.04, η^2^ = 0.157] and the interaction of IES-R category x coping strategy, *F*_(2,1160)_ = 23.48, η^2^ = 0.011, all *p* < 0.001. *Post-hoc* analysis indicated significant differences for all within and between factor comparisons (all *p* < 0.001), with those in the *PTSD* category scoring higher than the *Normal* category and with the EF coping strategy being higher than the PF, which in turn was higher than the DC coping strategy (Figure 4A).

In regards to the average scores reported for the EFA-identified factors (*TrnsV, CovMed, EdRout, IntF, AnxApp*, and *ChngLife*) relating to COVID-19-related sources of stress, across IES-R category (*Normal* vs. *PTSD*), analysis indicated a significant difference across IES-R category [*F*_(1,3030)_ = 272.80, η^2^ = 0.129], source of stress factor [*F*_(5,3030)_ = 143.15, η^2^ = 0.104] and the interaction of IES-R category x stress factor, *F*_(5,3030)_ = 6.02, η^2^ = 0.004, all *p* < 0.001. *Post-hoc* analysis indicated a significantly higher average score in the *PTSD* subgroup (interpreted as higher likelihood of perceiving the stressors as more stressful) relative to the *Normal* subgroup for all EFA-identified source of stress factors (all *p* < 0.001). In relation to the stress factors for the *Normal* IES-R category, all *p* < 0.001 except for *TrnsV* vs. *CovMed* and *AnxApp* vs. *ChngLife, p* < 0.01, while *CovMed* vs. *AnxApp, p* < 0.05. For the *PTSD* subgroup, both *IntF* and *EdRout* were significantly higher (all *p* < 0.001) than all other factors except relative to each other (*p* > 0.05), and both *CovMed* and *TrnsV* were significantly higher than *AnxApp, p* < 0.001 and *p* < 0.01, respectively. All other comparisons were not significant (*p* > 0.05) (Figure 4B).

## Discussion

The relationship that exists between a stressful event and the resultant impact on the mental health of an individual is complex. The complexity stems from the nature of the stressful event itself, in addition to how the individual perceives the stress and its consequences (threat vs. challenge), and how they ultimately respond to the event (emotional vs. logical) (80, 105).

As indicated above, we proposed that based on previous findings, coping strategies (emotion-focused, problem-focused, dysfunctional coping) would have a mediating effect on the relationship between the impact of the COVID-19 pandemic (stressor) and mental health (outcome). In our study, this investigation was conducted utilizing a cross-sectional methodology. As addressed earlier, the use of a cross-sectional study provides an opportunity to investigate the potential effects when the event is still taking place or shortly after, avoiding the potential for recall bias (29).

### COVID-19 Pandemic and Depression, Anxiety, and Stress

Our findings indicate a substantial proportion of students in the severe and extremely severe categories of the depression, anxiety and stress scales. In relation to the average scores, females scored higher than males in all mental health measures, significantly in anxiety and stress, and a tendency toward significance in depression. Additionally, a significant proportion of students (~50%) reported perceiving a deterioration in their mental health relative to before the onset of the COVID-19 pandemic, with the proportion of females being significantly higher than males.

### COVID-19 Pandemic: Impact, Coping, and Mental Health

In relation to the impact of the COVID-19 pandemic, a substantial proportion of students reported scores within the previously established cutoff values for PTSD, with a significantly higher proportion of females scoring at a level where PTSD is a clinical concern (100–102). Alarmingly, a significant percentage of females (~15%) fell in the highest category, which has been described as potentially impacting the immune system long-term (100). This proportion was also significantly higher than the proportion of males in the same category. These results, indicating levels of potential clinical concern within our sample, appear to also correspond with previous reports describing COVID-19 as a traumatic stressor resulting in PTSD-like responses (51). Moreover, all three IES-R subscales (AVD, INT, HYP) were significantly positively correlated to the three mental health outcomes (D, A, S). Additionally, all three IES-R subscales were most strongly correlated to the *dysfunctional coping* strategy of the Brief COPE, which was also the coping strategy that was most strongly related to the three mental health measures. Mediation analysis indicated that *dysfunctional coping* mediated the relationship between both AVD and HYP, and all three mental health measures. Additionally, the only mediation involving positive coping strategies [PF, EF; (82–86)] was that of *emotion-focused* coping mediating the relationship between AVD and depression, corroborating previous findings (106, 107). This is despite the fact that both the average scores of the positive coping mechanisms and the number of respondents reporting utilizing these coping mechanisms were higher than the DC strategy.

Pertaining to EF and PF (positive coping strategies), in addition to the general absence of a positive influence of such coping mechanisms on mental health as indicated by the mediation analysis, small positive relationships were observed in relation to the anxiety and stress scores. This may result from the potential for even positive coping mechanisms (e.g., finding comfort in religion or spiritual beliefs) to be misused (e.g., misuse of humor, spiritual bypass, etc.) (108–111) with the potential for negative mental health consequences.

Taken in the context of the data pertaining to the impact of the event (IES-R) indicating that ~33% of our females and 12% of our males were in a category of concern for the IES-R, and the rather substantial correlations between the IES-R and mental health (DASS-21) scores addressed above, it appears that coping strategies, while playing a role in mediating negative mental health outcomes through *dysfunctional coping* strategies, were not influential in minimizing the impact of the event on mental health. This may be indicative of potential additional factors/variables that may influence mental health outcomes more potently than the buffering capacity of the coping strategies addressed in this study.

### Chronic Stress: Physiology, Behavior, and Influence on Coping Responses

A potential major factor that may impact this dynamic between the stressor, the coping mechanism and the mental health outcome is chronic stress (as distinct from acute stress). Chronic stress is known to significantly influence physiology (112–114), including neurophysiology (115–117) and the immune system (118–120). As a result, it is to be expected that health in general will also be impacted. Additionally, and related, chronic stress contributes to the potential for an increased predisposition to psychiatric disorders such as depression and anxiety (121, 122). Ironically, it may also impact the efficacy of treatments (e.g., psychotropics, vaccinations, etc.) targeted toward the very same systems (e.g., central nervous system, immune system, etc.) affected by chronic stress (119, 123, 124).

Thus, given the complex impact of chronic stress, including at the neurophysiological level and its implication at the behavioral level (113, 116), it is reasonable to expect interference with the very behaviors that have the potential to be used to buffer the impact of stressful events. The response and coping strategies utilized in the presence of a stressor will vary and are dependent on the nature, duration and intensity of the stressor and the impact of the lingering stressor on the physiology. This dynamic may lead to a diversity of responses in the various systems (e.g., behavioral, endocrine) within the organism (125) that inform future responses. It is possible that part of this diversity of responses may involve a failure of healthy coping strategies (82) to positively impact mental health and the potential adoption of maladaptive coping strategies (126). This may potentially contribute to explaining the observations described above relating to the higher reported use of positive coping strategies in our sample with minimal impact on mental health outcomes.

Additionally, this may represent a physiological and psychological burnout that perpetuates further maladaptive coping and negative mental health (127, 128). This may reflect the observed relationship between *dysfunctional coping* and all three mental health parameters, in addition to the mediating role of dysfunctional coping in influencing the impact of the COVID-19 pandemic on mental health. Moreover, our results also indicate a substantial relationship between *dysfunctional coping* and what Mohr et al. refer to as defeatism (constituting a substantial portion of the *dysfunctional coping* questions of the Brief COPE). Defeatism, defined as consisting of such behaviors “like ‘giving up', ‘using alcohol' or ‘refusing to believe that this happened',” also reflects insufficient coping behavior in the presence of chronic stress (103, 104). In this regard, our data revealed a similar proportion of students displaying insufficient coping to those reported previously, cross-culturally, under chronic stress (104).

### COVID-19-Related Interventions and Impact

In relation to the COVID-19 pandemic, implemented secondary measures seeking to putatively address the pandemic (e.g., lockdowns, isolation, etc.) and the resulting consequences have the potential to influence how the event itself impacts the dynamic described above in relation to chronic stress, coping and mental health and the magnitude of such an impact. Such consequences have been reported extensively in other reports including, but not limited to, increased substance use to cope with the pandemic (46, 129), increased numbers of reported drug overdoses and related deaths (130–133), increased reports of suicidal ideation/thoughts/consideration of suicide (46, 47), loss of work/increased unemployment (134), financial hardships (135), loss of essential social interaction (40, 135, 136), physical health disturbances [e.g., sleep, exercise, eating habits; (47, 137–141)] and disruption of education (142).

Our results indicate that university students were not immune or exempt from the impact of the secondary measures implemented and discussed above. This is of special concern given that university students are within the age range (mid-teens to mid-20's) when a majority of mental disorders manifest themselves (143). Students are not shielded entirely from the realities of the events taking place around them, such as steps taken by various authorities (e.g., federal and local governments, school administrations etc.), and recognize the potential impact that such events have on their personal lives and the lives of those close to them (e.g., situations of unemployment). These experiences assist them in forming schemas informing their interpretation and perception of the same events.

In this regard, interestingly, pertaining to the secondary measures implemented in relation to COVID-19, the top 10 stressors reported by the student sample (sexes combined) reflected all the variables identified within the *Interference with Freedom* and *Education Routine* factors, in addition to two variables within the *Transmission of COVID-19 Virus* factor, specifically the “Possibility of interruption of my life because of testing positive” and the “Possibility of interruption of my life because of someone close testing positive.” All of these variables reflect an interruption of life by the measures implemented, rather than concerns directly relating to the actual SARS-CoV-2 virus itself, including the potential for contracting it.

### Limitations and Future Research

Given the complexity of the events that have occurred since the outbreak of COVID-19, the global nature of the event, the complexity of human behavior and the potential uniqueness of the sample involving students from a single location, caution is necessary, as is in fact with all human studies, in extrapolating and generalizing the findings and subsequent implications. Related is the complication associated with more localized restrictions, at the state, the city and even at the institutional level. Moreover, the findings of this study need to be interpreted in the context of the large body of research that continues to accumulate addressing the indirect impact of the COVID-19 pandemic associated with the measures taken. However, consideration also needs to be given to the consistencies of our results with cross-cultural, cross-age and cross-sex findings reported in both national and international studies. A limitation of our work is the potential for survey fatigue due to the length of the survey. Associated with this limitation is the logistical constraint present in any survey research on the number of topics investigated within a single survey. Additionally, in relation to coping strategies, while the 14 Brief COPE subscales addressed a number of coping mechanisms (e.g., the use of religion/faith for emotion-focused coping) constituting the three principal categories (EF, PF, and DC), they were limited in their capacity to address these in great depth, e.g., positive vs. negative religious coping. Our study also did not provide an opportunity for the participants to report how they personally appraised the event. Future research should take into consideration these limitations in addition to considering addressing in greater depth the potential for differential impacts on mental health of the various interventions implemented in different institutions, localities, states and countries.

## Conclusion

In conclusion, our study appears to reflect a minimal impact of the positive coping mechanisms investigated (emotion-focused, problem-focused) in minimizing the negative mental health outcomes in the context of the COVID-19 pandemic. This was evident in the higher levels of reported use of such strategies, but with minimal mediation of any of the mental health parameters. Conversely, while the use of *dysfunctional coping* mediated the relationship between the impact of the event and mental health, this was also the coping mechanism reported to be used least by the students. Given (1) the absence of an impact of the positive coping mechanisms investigated, which have been previously reported to positively influence mental health, (2) the higher prevalence of the utilization of positive coping mechanisms relative to the *dysfunctional coping* strategy, (3) the perceived worsening of mental health relative to prior to the pandemic, (4) the reported significant COVID-19-related stressors, and (5) previous non-COVID-19-related research [e.g., (82)] indicating the potential that emotion- and problem-focused strategies are effective in “benign” but not “severe” events, our study appears to highlight an influence of external factors that may override the capacity/efficacy of certain coping mechanisms (e.g., those measured in this study) to positively impact mental health in the presence of a significant chronic stressor. Our results cannot be interpreted as concluding that other positive coping mechanisms may not potentially play a role in mediating the stress imposed by the implemented measures and restrictions during the COVID-19 pandemic. Rather, the implication is that, in light of the reviewed literature indicating the specific positive coping strategies addressed as mediators in this study (EF and PF) in reducing the impact of various stressors on mental health, it appears, and it should be of concern, that the stress resulting from the putative mitigation efforts significantly overwhelmed the mediating capacity of these specific coping measures. Moreover, our results appear to corroborate previous literature indicating the potential exacerbation of negative mental health issues, including PTSD-like reactions, by the COVID-19 pandemic (51) and show a similarity in impact on mental health to those observed in previous major events (e.g., SARS-CoV-1, September 11, 2001 terrorist attacks in the US), as well as other reports relating to the current COVID-19 pandemic, reflecting significant negative mental health consequences in the general population, including in university students (29–31, 33, 34, 51, 144–147).

Our research indicates the necessity for continued surveillance of mental health in relation to the ongoing COVID-19 pandemic and the associated interventions within the university student population. This includes the need for efforts to reduce the impact of influences that may contribute to propagating a sense of despondency (55), potentially amplifying behaviors that may result from or increase the predisposition to negative mental health outcomes, such as alcohol consumption, drug abuse, suicidal ideation, etc. (47, 53, 131–133, 148). Efforts should also target measures that ensure the presence of sufficient supporting elements (e.g., counseling centers and staff), in addition to educational efforts that develop and facilitate personality traits that build strength in character. These would, in turn, support the development of positive mental health characteristics and the ability to look beyond the perceived threat and recognize and utilize stressful events/adversity as a challenge enabling growth. Such efforts would assist in potentially avoiding the implications of the response to major stressful events being primarily controlled by fear with minimal involvement of reason (logical response) [17, 80, 104]. However, there is a major caveat to this, that is very pertinent to the current COVID-19 pandemic, in regard to how successful and beneficial the above measures could be based on the personal appraisal (challenge vs. threat) of the event by an individual. As previously reported, events that are “high in personal significance and low in controllability” are considered as being a threat (149), and events perceived as a threat may lead to a dysfunctional response. This becomes a grave concern when a significant body of literature pertaining to the impact of the COVID-19 pandemic at the psychological level (including our findings in university students) appears to indicate a predominant global appraisal of the COVID-19 pandemic and related interventions as a threat.

Thus, while this study specifically pertained to the university student population and the COVID-19 pandemic, the implications potentially impact the broader population and events other than the COVID-19 pandemic. Given the lack of a role of positive coping, attention needs to be given to the factors that are outside the individual's control, and the potential role that the factors may play in being sources of stress. Our findings appear to highlight the need for a more integrated psycho-neuro-endocrinological approach that considers the broader well-being of society and avoids the potential significant detrimental ramifications of a reactionary myopic approach to events such as the COVID-19 pandemic, in order to truly and best serve the common good of humanity.

## Data Availability Statement

The raw data supporting the conclusions of this article will be made available by the authors, without undue reservation, to any qualified researcher.

## Ethics Statement

The studies involving human participants were reviewed and approved by Franciscan University of Steubenville Institutional Review Board (IRB; #2020-08). The patients/participants provided their written informed consent to participate in this study.

## Author Contributions

SS supervised the study. SS and CC contributed to the conception, design of the study, conducting of the study, and contributed to manuscript revision. SS, CC, CF, and KO'B performed the statistical analyses and contributed to the writing of the first draft of the manuscript. All authors read and approved the submitted version.

## Funding

Funding for this study was provided by Franciscan University of Steubenville. The funders had no role in study design, data collection and analysis, interpretation of results, decision to publish or preparation of the manuscript.

## Conflict of Interest

The authors declare that the research was conducted in the absence of any commercial or financial relationships that could be construed as a potential conflict of interest.

## Publisher's Note

All claims expressed in this article are solely those of the authors and do not necessarily represent those of their affiliated organizations, or those of the publisher, the editors and the reviewers. Any product that may be evaluated in this article, or claim that may be made by its manufacturer, is not guaranteed or endorsed by the publisher.
