# Supplementary material for: The Impact of COVID-19 and Associated Interventions on Mental Health: A Cross-Sectional Study in a Sample of University Students
Source: Front Psychiatry. 2022 Jan 26;12:801859. doi: 10.3389/fpsyt.2021.801859 (PMC8825780; doi:10.3389/fpsyt.2021.801859)
Supplement: Supplementary file 1 [file Table_1.DOCX]

**Supplementary Table 1.** Complete results for mediation pathways between IES-R subscales (HYP, INT, AVD) and Depression with Brief COPE subscales (EF, PF, DC) as potential mediators. *HYP*: hyperarousal, *INT*: intrusion, *AVD*: avoidance; *EF*: emotion-focused, *PF*: problem-focused, *DC*: dysfunctional coping.

| Indirect and Total Effects | | | | | | | | | | | | | | | | | |
| --- | --- | --- | --- | --- | --- | --- | --- | --- | --- | --- | --- | --- | --- | --- | --- | --- | --- |
|  | | | | | | | | **95% C.I. (a)** | | | |  | | | | | |
| **Type** | | **Effect** | | **Estimate** | | **SE** | | **Lower** | | **Upper** | | **β** | | **z** | | **p** | |
| Indirect |  | HYP ⇒ PF ⇒ D |  | -0.1048 |  | 0.1182 |  | -0.3977 |  | 0.0540 |  | -0.00793 |  | -0.886 |  | 0.376 |  |
|  |  | HYP ⇒ EF ⇒ D |  | 0.0788 |  | 0.1699 |  | -0.2154 |  | 0.4515 |  | 0.00596 |  | 0.464 |  | 0.643 |  |
|  |  | HYP ⇒ DC ⇒ D |  | 2.0106 |  | 0.5156 |  | 1.0974 |  | 3.1201 |  | 0.15221 |  | 3.899 |  | < .001 |  |
|  |  | INT ⇒ PF ⇒ D |  | -0.3390 |  | 0.2152 |  | -0.7847 |  | 0.0595 |  | -0.02513 |  | -1.575 |  | 0.115 |  |
|  |  | INT ⇒ EF ⇒ D |  | -0.2145 |  | 0.1800 |  | -0.5962 |  | 0.1150 |  | -0.01590 |  | -1.192 |  | 0.233 |  |
|  |  | INT ⇒ DC ⇒ D |  | 0.3181 |  | 0.4856 |  | -0.6148 |  | 1.3292 |  | 0.02358 |  | 0.655 |  | 0.513 |  |
|  |  | AVD ⇒ PF ⇒ D |  | 0.0557 |  | 0.0760 |  | -0.0659 |  | 0.2398 |  | 0.00478 |  | 0.733 |  | 0.463 |  |
|  |  | AVD ⇒ EF ⇒ D |  | -0.3252 |  | 0.1451 |  | -0.6376 |  | -0.0772 |  | -0.02788 |  | -2.241 |  | 0.025 |  |
|  |  | AVD ⇒ DC ⇒ D |  | 2.1388 |  | 0.3379 |  | 1.4983 |  | 2.8174 |  | 0.18340 |  | 6.330 |  | < .001 |  |
| Component |  | HYP ⇒ PF |  | 0.0974 |  | 0.0779 |  | -0.0478 |  | 0.2563 |  | 0.09949 |  | 1.250 |  | 0.211 |  |
|  |  | PF ⇒ D |  | -1.0753 |  | 0.6345 |  | -2.2987 |  | 0.1900 |  | -0.07972 |  | -1.695 |  | 0.090 |  |
|  |  | HYP ⇒ EF |  | -0.0313 |  | 0.0636 |  | -0.1612 |  | 0.0846 |  | -0.03953 |  | -0.492 |  | 0.622 |  |
|  |  | EF ⇒ D |  | -2.5145 |  | 0.7731 |  | -4.0436 |  | -0.9519 |  | -0.15085 |  | -3.253 |  | 0.001 |  |
|  |  | HYP ⇒ DC |  | 0.2049 |  | 0.0485 |  | 0.1177 |  | 0.3067 |  | 0.31598 |  | 4.223 |  | < .001 |  |
|  |  | DC ⇒ D |  | 9.8143 |  | 1.0430 |  | 7.7940 |  | 11.9537 |  | 0.48170 |  | 9.409 |  | < .001 |  |
|  |  | INT ⇒ PF |  | 0.3153 |  | 0.0813 |  | 0.1532 |  | 0.4730 |  | 0.31521 |  | 3.876 |  | < .001 |  |
|  |  | INT ⇒ EF |  | 0.0853 |  | 0.0649 |  | -0.0480 |  | 0.2129 |  | 0.10540 |  | 1.314 |  | 0.189 |  |
|  |  | INT ⇒ DC |  | 0.0324 |  | 0.0484 |  | -0.0650 |  | 0.1285 |  | 0.04894 |  | 0.670 |  | 0.503 |  |
|  |  | AVD ⇒ PF |  | -0.0518 |  | 0.0561 |  | -0.1630 |  | 0.0514 |  | -0.05996 |  | -0.924 |  | 0.356 |  |
|  |  | AVD ⇒ EF |  | 0.1293 |  | 0.0400 |  | 0.0557 |  | 0.2062 |  | 0.18483 |  | 3.233 |  | 0.001 |  |
|  |  | AVD ⇒ DC |  | 0.2179 |  | 0.0334 |  | 0.1539 |  | 0.2803 |  | 0.38073 |  | 6.529 |  | < .001 |  |
| Direct |  | HYP ⇒ D |  | 4.8627 |  | 0.9663 |  | 2.7634 |  | 6.5411 |  | 0.36811 |  | 5.032 |  | < .001 |  |
|  |  | INT ⇒ D |  | -1.8736 |  | 0.9713 |  | -3.6558 |  | 0.0857 |  | -0.13888 |  | -1.929 |  | 0.054 |  |
|  |  | AVD ⇒ D |  | 0.6233 |  | 0.6299 |  | -0.5901 |  | 1.9277 |  | 0.05345 |  | 0.990 |  | 0.322 |  |
| Total |  | HYP ⇒ D |  | 6.8473 |  | 0.8416 |  | 5.1978 |  | 8.4969 |  | 0.52426 |  | 8.136 |  | < .001 |  |
|  |  | INT ⇒ D |  | -2.1090 |  | 0.8833 |  | -3.8402 |  | -0.3778 |  | -0.15811 |  | -2.388 |  | 0.017 |  |
|  |  | AVD ⇒ D |  | 2.4927 |  | 0.5624 |  | 1.3903 |  | 3.5950 |  | 0.21618 |  | 4.432 |  | < .001 |  |
| Note. Confidence intervals computed with method: Bootstrap percentiles | | | | | | | | | | | | | | | | | |
| Note. Betas are completely standardized effect sizes | | | | | | | | | | | | | | | | | |
|  | | | | | | | | | | | | | | | | | |
