# Supplementary material for: The Impact of COVID-19 and Associated Interventions on Mental Health: A Cross-Sectional Study in a Sample of University Students
Source: Front Psychiatry. 2022 Jan 26;12:801859. doi: 10.3389/fpsyt.2021.801859 (PMC8825780; doi:10.3389/fpsyt.2021.801859)
Supplement: Supplementary file 2 [file Table_2.DOCX]

**Supplementary Table 2.** Complete results for mediation pathways between IES-R subscales (HYP, INT, AVD) and Anxiety with Brief COPE subscales (EF, PF, DC) as potential mediators. *HYP*: hyperarousal, *INT*: intrusion, *AVD*: avoidance; *EF*: emotion-focused, *PF*: problem-focused, *DC*: dysfunctional coping.

| Indirect and Total Effects | | | | | | | | | | | | | | | | | |
| --- | --- | --- | --- | --- | --- | --- | --- | --- | --- | --- | --- | --- | --- | --- | --- | --- | --- |
|  | | | | | | | | **95% C.I. (a)** | | | |  | | | | | |
| **Type** | | **Effect** | | **Estimate** | | **SE** | | **Lower** | | **Upper** | | **β** | | **z** | | **p** | |
| Indirect |  | HYP ⇒ PF ⇒ A |  | -0.0633 |  | 0.0841 |  | -0.2831 |  | 0.0510 |  | -0.00577 |  | -0.753 |  | 0.452 |  |
|  |  | HYP ⇒ EF ⇒ A |  | 0.0130 |  | 0.0526 |  | -0.0655 |  | 0.1584 |  | 0.00118 |  | 0.246 |  | 0.805 |  |
|  |  | HYP ⇒ DC ⇒ A |  | 0.8029 |  | 0.2800 |  | 0.3477 |  | 1.4840 |  | 0.07323 |  | 2.868 |  | 0.004 |  |
|  |  | INT ⇒ PF ⇒ A |  | -0.2048 |  | 0.1703 |  | -0.5619 |  | 0.1240 |  | -0.01829 |  | -1.202 |  | 0.229 |  |
|  |  | INT ⇒ EF ⇒ A |  | -0.0353 |  | 0.0746 |  | -0.2400 |  | 0.0766 |  | -0.00315 |  | -0.473 |  | 0.636 |  |
|  |  | INT ⇒ DC ⇒ A |  | 0.1270 |  | 0.2030 |  | -0.2452 |  | 0.5570 |  | 0.01134 |  | 0.626 |  | 0.532 |  |
|  |  | AVD ⇒ PF ⇒ A |  | 0.0337 |  | 0.0480 |  | -0.0388 |  | 0.1441 |  | 0.00348 |  | 0.702 |  | 0.483 |  |
|  |  | AVD ⇒ EF ⇒ A |  | -0.0535 |  | 0.0834 |  | -0.2379 |  | 0.1118 |  | -0.00553 |  | -0.642 |  | 0.521 |  |
|  |  | AVD ⇒ DC ⇒ A |  | 0.8540 |  | 0.2330 |  | 0.4043 |  | 1.3273 |  | 0.08824 |  | 3.666 |  | < .001 |  |
| Component |  | HYP ⇒ PF |  | 0.0974 |  | 0.0794 |  | -0.0544 |  | 0.2500 |  | 0.09949 |  | 1.226 |  | 0.220 |  |
|  |  | PF ⇒ A |  | -0.6495 |  | 0.5173 |  | -1.6623 |  | 0.3626 |  | -0.05802 |  | -1.256 |  | 0.209 |  |
|  |  | HYP ⇒ EF |  | -0.0313 |  | 0.0646 |  | -0.1601 |  | 0.0928 |  | -0.03953 |  | -0.485 |  | 0.628 |  |
|  |  | EF ⇒ A |  | -0.4138 |  | 0.6022 |  | -1.6157 |  | 0.8407 |  | -0.02991 |  | -0.687 |  | 0.492 |  |
|  |  | HYP ⇒ DC |  | 0.2049 |  | 0.0498 |  | 0.1158 |  | 0.3054 |  | 0.31598 |  | 4.115 |  | < .001 |  |
|  |  | DC ⇒ A |  | 3.9189 |  | 1.0085 |  | 1.9325 |  | 5.9436 |  | 0.23176 |  | 3.886 |  | < .001 |  |
|  |  | INT ⇒ PF |  | 0.3153 |  | 0.0804 |  | 0.1558 |  | 0.4686 |  | 0.31521 |  | 3.922 |  | < .001 |  |
|  |  | INT ⇒ EF |  | 0.0853 |  | 0.0646 |  | -0.0320 |  | 0.2146 |  | 0.10540 |  | 1.321 |  | 0.187 |  |
|  |  | INT ⇒ DC |  | 0.0324 |  | 0.0493 |  | -0.0644 |  | 0.1264 |  | 0.04894 |  | 0.658 |  | 0.511 |  |
|  |  | AVD ⇒ PF |  | -0.0518 |  | 0.0524 |  | -0.1542 |  | 0.0524 |  | -0.05996 |  | -0.989 |  | 0.323 |  |
|  |  | AVD ⇒ EF |  | 0.1293 |  | 0.0397 |  | 0.0528 |  | 0.2059 |  | 0.18483 |  | 3.254 |  | 0.001 |  |
|  |  | AVD ⇒ DC |  | 0.2179 |  | 0.0328 |  | 0.1576 |  | 0.2846 |  | 0.38073 |  | 6.649 |  | < .001 |  |
| Direct |  | HYP ⇒ A |  | 4.2241 |  | 0.9955 |  | 2.2126 |  | 6.1998 |  | 0.38529 |  | 4.243 |  | < .001 |  |
|  |  | INT ⇒ A |  | 0.2844 |  | 0.8880 |  | -1.5351 |  | 2.0574 |  | 0.02540 |  | 0.320 |  | 0.749 |  |
|  |  | AVD ⇒ A |  | 1.0923 |  | 0.5866 |  | -0.0177 |  | 2.3164 |  | 0.11286 |  | 1.862 |  | 0.063 |  |
| Total |  | HYP ⇒ A |  | 4.9766 |  | 0.6670 |  | 3.6692 |  | 6.2840 |  | 0.45493 |  | 7.461 |  | < .001 |  |
|  |  | INT ⇒ A |  | 0.1714 |  | 0.7001 |  | -1.2007 |  | 1.5435 |  | 0.01534 |  | 0.245 |  | 0.807 |  |
|  |  | AVD ⇒ A |  | 1.9265 |  | 0.4458 |  | 1.0528 |  | 2.8002 |  | 0.19948 |  | 4.322 |  | < .001 |  |
| Note. Confidence intervals computed with method: Bootstrap percentiles | | | | | | | | | | | | | | | | | |
| Note. Betas are completely standardized effect sizes | | | | | | | | | | | | | | | | | |
|  | | | | | | | | | | | | | | | | | |
