# Supplementary material for: The Impact of COVID-19 and Associated Interventions on Mental Health: A Cross-Sectional Study in a Sample of University Students
Source: Front Psychiatry. 2022 Jan 26;12:801859. doi: 10.3389/fpsyt.2021.801859 (PMC8825780; doi:10.3389/fpsyt.2021.801859)
Supplement: Supplementary file 3 [file Table_3.DOCX]

**Table 3.** Complete results for mediation pathways between IES-R subscales (HYP, INT, AVD) and Stress with Brief COPE subscales (EF, PF, DC) as potential mediators. *HYP*: hyperarousal, *INT*: intrusion, *AVD*: avoidance; *EF*: emotion-focused, *PF*: problem-focused, *DC*: dysfunctional coping.

| Indirect and Total Effects | | | | | | | | | | | | | | | | | |
| --- | --- | --- | --- | --- | --- | --- | --- | --- | --- | --- | --- | --- | --- | --- | --- | --- | --- |
|  | | | | | | | | **95% C.I. (a)** | | | |  | | | | | |
| **Type** | | **Effect** | | **Estimate** | | **SE** | | **Lower** | | **Upper** | | **β** | | **z** | | **p** | |
| Indirect |  | HYP ⇒ PF ⇒ S |  | -0.00602 |  | 0.0751 |  | -0.1901 |  | 0.1327 |  | -4.69e−4 |  | -0.0802 |  | 0.936 |  |
|  |  | HYP ⇒ EF ⇒ S |  | 0.02180 |  | 0.0580 |  | -0.0936 |  | 0.1530 |  | 0.00170 |  | 0.3759 |  | 0.707 |  |
|  |  | HYP ⇒ DC ⇒ S |  | 1.23774 |  | 0.3713 |  | 0.5884 |  | 2.0624 |  | 0.09642 |  | 3.3331 |  | < .001 |  |
|  |  | INT ⇒ PF ⇒ S |  | -0.01948 |  | 0.1854 |  | -0.3958 |  | 0.3496 |  | -0.00149 |  | -0.1051 |  | 0.916 |  |
|  |  | INT ⇒ EF ⇒ S |  | -0.05935 |  | 0.0785 |  | -0.2375 |  | 0.0764 |  | -0.00453 |  | -0.7557 |  | 0.450 |  |
|  |  | INT ⇒ DC ⇒ S |  | 0.19579 |  | 0.3135 |  | -0.3933 |  | 0.8720 |  | 0.01493 |  | 0.6245 |  | 0.532 |  |
|  |  | AVD ⇒ PF ⇒ S |  | 0.00320 |  | 0.0421 |  | -0.0863 |  | 0.1019 |  | 2.83e-4 |  | 0.0760 |  | 0.939 |  |
|  |  | AVD ⇒ EF ⇒ S |  | -0.08997 |  | 0.0902 |  | -0.2744 |  | 0.0861 |  | -0.00794 |  | -0.9972 |  | 0.319 |  |
|  |  | AVD ⇒ DC ⇒ S |  | 1.31661 |  | 0.2708 |  | 0.7970 |  | 1.8669 |  | 0.11617 |  | 4.8623 |  | < .001 |  |
| Component |  | HYP ⇒ PF |  | 0.09743 |  | 0.0795 |  | -0.0513 |  | 0.2503 |  | 0.09949 |  | 1.2258 |  | 0.220 |  |
|  |  | PF ⇒ S |  | -0.06181 |  | 0.5723 |  | -1.2448 |  | 1.0636 |  | -0.00471 |  | -0.1080 |  | 0.914 |  |
|  |  | HYP ⇒ EF |  | -0.03133 |  | 0.0635 |  | -0.1603 |  | 0.0897 |  | -0.03953 |  | -0.4937 |  | 0.621 |  |
|  |  | EF ⇒ S |  | -0.69576 |  | 0.6462 |  | -1.8383 |  | 0.6722 |  | -0.04295 |  | -1.0766 |  | 0.282 |  |
|  |  | HYP ⇒ DC |  | 0.20487 |  | 0.0496 |  | 0.1174 |  | 0.3060 |  | 0.31598 |  | 4.1298 |  | < .001 |  |
|  |  | DC ⇒ S |  | 6.04165 |  | 1.2122 |  | 3.5950 |  | 8.4739 |  | 0.30514 |  | 4.9839 |  | < .001 |  |
|  |  | INT ⇒ PF |  | 0.31525 |  | 0.0798 |  | 0.1644 |  | 0.4734 |  | 0.31521 |  | 3.9483 |  | < .001 |  |
|  |  | INT ⇒ EF |  | 0.08531 |  | 0.0632 |  | -0.0445 |  | 0.2058 |  | 0.10540 |  | 1.3488 |  | 0.177 |  |
|  |  | INT ⇒ DC |  | 0.03241 |  | 0.0499 |  | -0.0728 |  | 0.1262 |  | 0.04894 |  | 0.6499 |  | 0.516 |  |
|  |  | AVD ⇒ PF |  | -0.05184 |  | 0.0517 |  | -0.1481 |  | 0.0519 |  | -0.05996 |  | -1.0018 |  | 0.316 |  |
|  |  | AVD ⇒ EF |  | 0.12932 |  | 0.0404 |  | 0.0512 |  | 0.2117 |  | 0.18483 |  | 3.2010 |  | 0.001 |  |
|  |  | AVD ⇒ DC |  | 0.21792 |  | 0.0329 |  | 0.1553 |  | 0.2827 |  | 0.38073 |  | 6.6214 |  | < .001 |  |
| Direct |  | HYP ⇒ S |  | 5.43016 |  | 0.8835 |  | 3.5743 |  | 7.1402 |  | 0.42300 |  | 6.1465 |  | < .001 |  |
|  |  | INT ⇒ S |  | -1.17753 |  | 0.8838 |  | -2.9886 |  | 0.4815 |  | -0.08982 |  | -1.3324 |  | 0.183 |  |
|  |  | AVD ⇒ S |  | 1.35568 |  | 0.6652 |  | 0.0581 |  | 2.6692 |  | 0.11962 |  | 2.0380 |  | 0.042 |  |
| Total |  | HYP ⇒ S |  | 6.68368 |  | 0.7745 |  | 5.1658 |  | 8.2016 |  | 0.52187 |  | 8.6301 |  | < .001 |  |
|  |  | INT ⇒ S |  | -1.06057 |  | 0.8128 |  | -2.6537 |  | 0.5325 |  | -0.08109 |  | -1.3048 |  | 0.192 |  |
|  |  | AVD ⇒ S |  | 2.58552 |  | 0.5176 |  | 1.5711 |  | 3.5999 |  | 0.22868 |  | 4.9955 |  | < .001 |  |
| Note. Confidence intervals computed with method: Bootstrap percentiles | | | | | | | | | | | | | | | | | |
| Note. Betas are completely standardized effect sizes | | | | | | | | | | | | | | | | | |
|  | | | | | | | | | | | | | | | | | |
