# Supplementary figures and images for: The Impact of COVID-19 and Associated Interventions on Mental Health: A Cross-Sectional Study in a Sample of University Students
Source: Front Psychiatry. 2022 Jan 26;12:801859. doi: 10.3389/fpsyt.2021.801859 (PMC8825780; doi:10.3389/fpsyt.2021.801859)

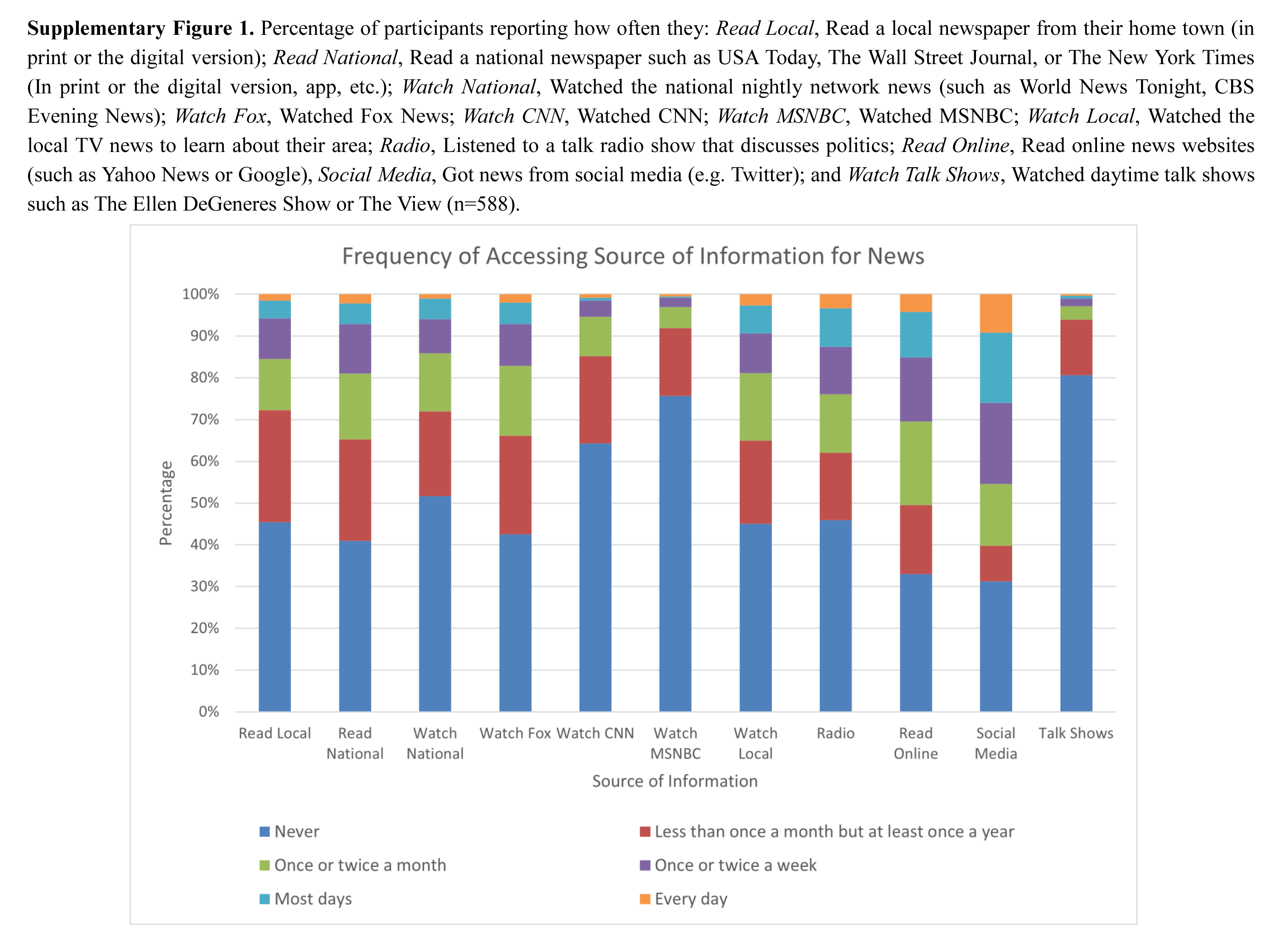

Supplement: Supplementary file 4 [file Image_1.TIF]

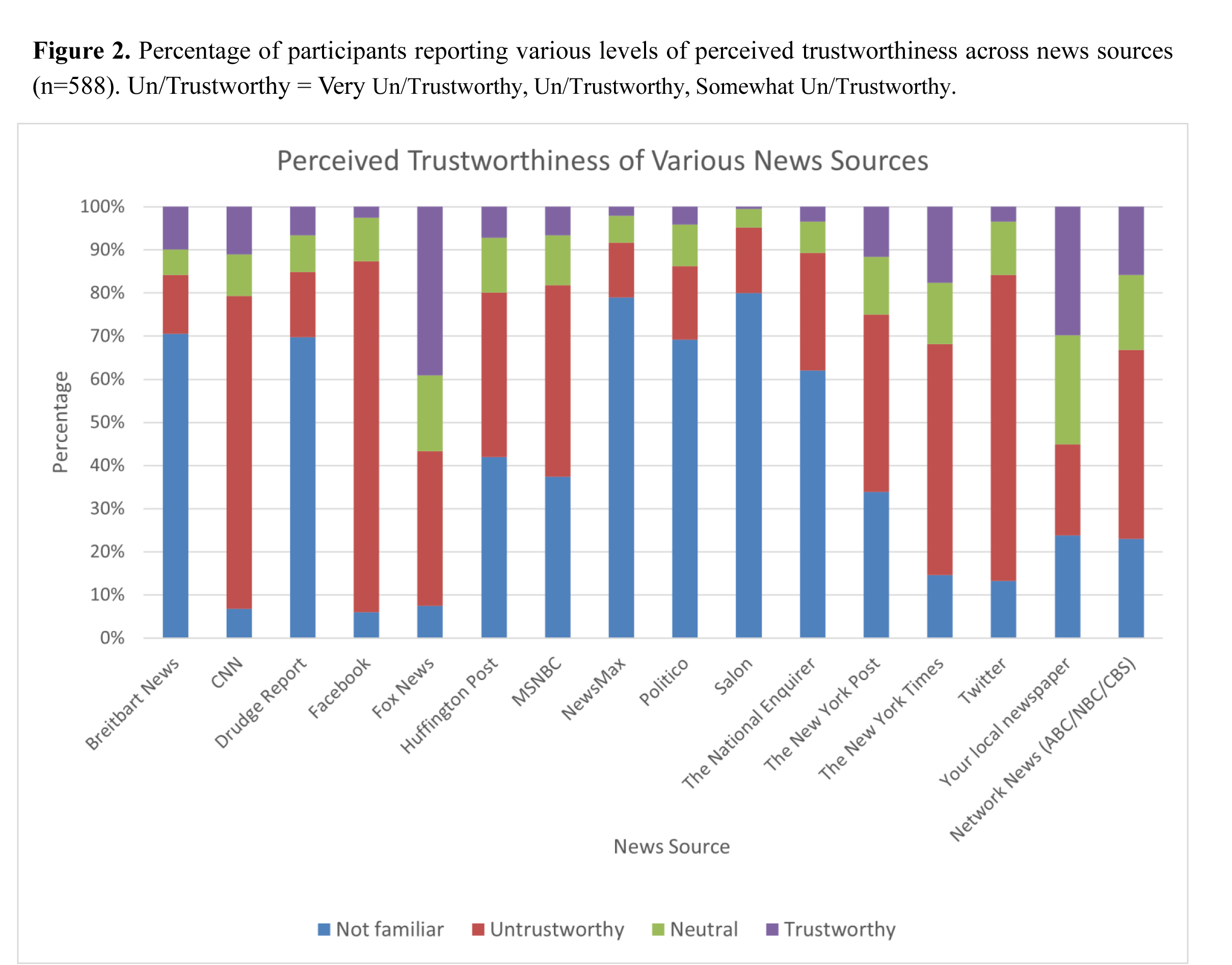

Supplement: Supplementary file 5 [file Image_2.TIF]
